# Recent Developments of Transition Metal Compounds-Carbon Hybrid Electrodes for High Energy/Power Supercapacitors

**DOI:** 10.1007/s40820-021-00642-2

**Published:** 2021-05-17

**Authors:** Kang Ren, Zheng Liu, Tong Wei, Zhuangjun Fan

**Affiliations:** State Key Laboratory of Heavy Oil Processing, School of Materials Science and Engineering, China University of Petroleum (East China), Qingdao, 266580 People’s Republic of China

**Keywords:** TMCs/carbon hybrid, Supercapacitors, High power density, Carbon skeleton, Interfacial engineering, Transition metal electronic structure

## Abstract

Due to their rapid power delivery, fast charging, and long cycle life, supercapacitors have become an important energy storage technology recently. However, to meet the continuously increasing demands in the fields of portable electronics, transportation, and future robotic technologies, supercapacitors with higher energy densities without sacrificing high power densities and cycle stabilities are still challenged. Transition metal compounds (TMCs) possessing high theoretical capacitance are always used as electrode materials to improve the energy densities of supercapacitors. However, the power densities and cycle lives of such TMCs-based electrodes are still inferior due to their low intrinsic conductivity and large volume expansion during the charge/discharge process, which greatly impede their large-scale applications. Most recently, the ideal integrating of TMCs and conductive carbon skeletons is considered as an effective solution to solve the above challenges. Herein, we summarize the recent developments of TMCs/carbon hybrid electrodes which exhibit both high energy/power densities from the aspects of structural design strategies, including conductive carbon skeleton, interface engineering, and electronic structure. Furthermore, the remaining challenges and future perspectives are also highlighted so as to provide strategies for the high energy/power TMCs/carbon-based supercapacitors.

## Highlights

- The development of transition metal compounds-carbon hybrid electrodes for high energy/power supercapacitors is summarized.
- Effects of the conductive carbon skeleton, interfacial engineering, and electronic structure for transition metal compounds-carbon hybrid are discussed.
- Some perspectives and issues in the future are provided.

## Graphical abstract

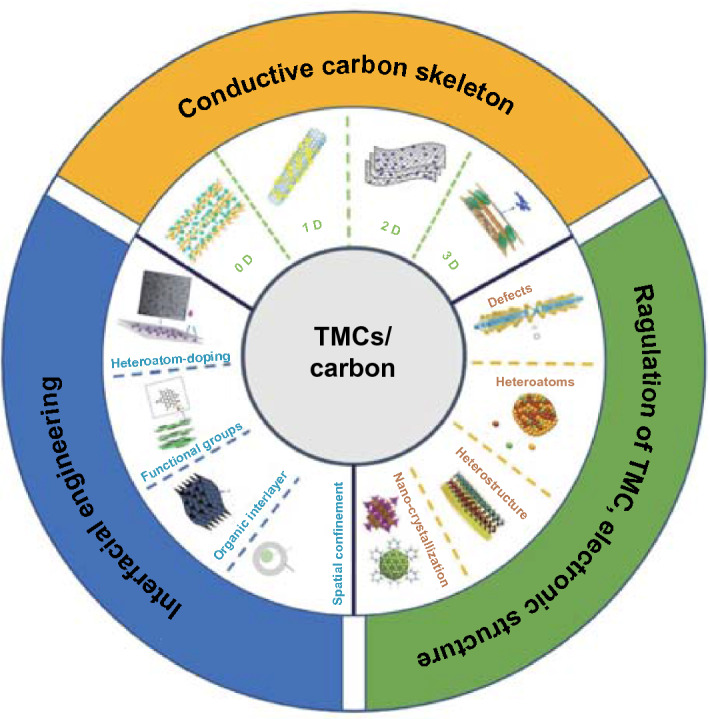

## Introduction

In recent years, the intense consumption of fossil energy and environmental pollution has brought great challenges to the survival and development of human beings. Therefore, searching for clean and sustainable energy sources has become one of the most important topics [1–4]. Especially for intermittent energy storage, such as wind energy, tidal energy, and solar energy, new technologies suited for both high energy density and power density are urgently needed [4–6]. Electrochemical capacitors commonly refer to as supercapacitors, which offer high power density, superior rate performance, and excellent cycle stability [7], have become a prime candidate for the high power applications that require fast on–off response such as hybrid electric vehicles, grid stabilization systems, forklifts, load cranes, aerospace equipment, and military weapons [6, 8]. Current commercial supercapacitors are mainly based on carbon materials, which store/release electrical charges through the physical adsorption/desorption of charges or electrolyte ions on the electrode surface, also called electrochemical double layer capacitors (EDLCs) [9]. Compared with commercial batteries (> 100 Wh kg^−1^), the energy densities of EDLCs (less than 10 Wh kg^−1^) are still low, which have become a great challenge for the large-scale application and further popularization of supercapacitors [10, 11]. To address the issue, the development of alternative electrode materials with higher specific capacitance is extremely urgent.

Transition metal compounds (TMCs), such as metal oxides, hydroxides, sulfides, and phosphides, have attracted great attention due to their high theoretical capacitance [12, 13], abundant sources, and low cost, etc. [14–16]. In this case, the studies of TMC-based supercapacitors are rising up due to their obviously enlarged energy densities (approaching 10^2^ Wh kg^−1^) [3]. The energy storage mechanisms of TMC-based electrodes primarily involve the electron transfer in the process of redox reactions and valence state changes of transition metal ions, indicating that the ion diffusion rate and phase transformation due to the change of valence state are important for electrochemical properties. Such transformation generally leads to the sluggish bulk phase reaction and dramatic volumetric expansion/shrinkage during the energy storage process and finally resulting in the low chemical reaction kinetics and inferior cycle stabilities of TMC-based supercapacitors. Since rapid charge/discharge capability and long cycling stability of minimum 10,000 cycles are key criteria for supercapacitors, the poor electrical conductivity, low power density, unsatisfactory mechanical/electrochemical stability, and some other problems, still limit their prospects of TMCs for large-scaled applications [17].

Due to the superior electric conductivity, large specific surface area, low cost, and structural controllability, carbon materials, such as graphene quantum dots, carbon nanotubes, graphene, porous carbon, etc., have been widely researched for energy storage and conversion [7, 18, 19]. Therefore, combining with various dimensional carbon skeletons can not only improve the intrinsic low conductivity, but also modify the morphology of transition metal crystals, which is beneficial for the enhancement of reaction kinetics, fast charging/discharging rate, and cycling stability of TMCs/carbon composites [20]. Although a large number of studies have been done for TMCs/carbon composites, the physical mixture or simple combination of transition metal derivative particles with carbon skeletons still faces some inevitable challenges, such as huge volume expansion/shrinkage during ultra-long charge/discharge process, or even breaking down of electrochemically active materials from carbon surface due to the vulnerable TMCs/carbon interfaces.

Recently, TMCs with nanoscale dimensions, especially quantum dot morphology, have aroused enormous attention in various applications because of their attractive intrinsic characteristics, such as small size effect, quantum tunneling effect, coulomb blockade effect, and surface effect [21–23]. Compared with bulk materials, the combination of nanoscale TMCs and carbon skeletons can provide improved electrochemical performance, which is beneficial for the construction of fast ions transport [24, 25]. In addition to nano-crystallization, the rational modification of TMCs crystals, such as the introduction of lattice defects, heterostructure, metal heteroatoms, etc., can also certainly enhance the redox reaction kinetics through decreasing reaction energy or shortening the ions diffusion paths [16]. However, nanoscale TMCs, which show high surface area/energy and large amounts of surface electrons, unavoidably aggregate during the long-times charge/discharge process. Therefore, how to keep the uniform dispersion and stable presence of nanoscale TMCs is another vital challenge that needs to be urgently solved. The construction of interfacial adhesion between TMCs and carbon skeletons may be an effective strategy to promote the uniform and stable dispersion of nanoscale-TMCs on the carbon surface. While it is crucial to systematically understand the design principles from the aspects of carbon skeletons, TMC’s electronic structure, and interfaces for TMCs/carbon composites, there is a lack of review that dedicates to the up-to-date reports for boosting the power/energy densities and cycling stabilities of TMCs/carbon-based supercapacitors.

**Fig. 1:**
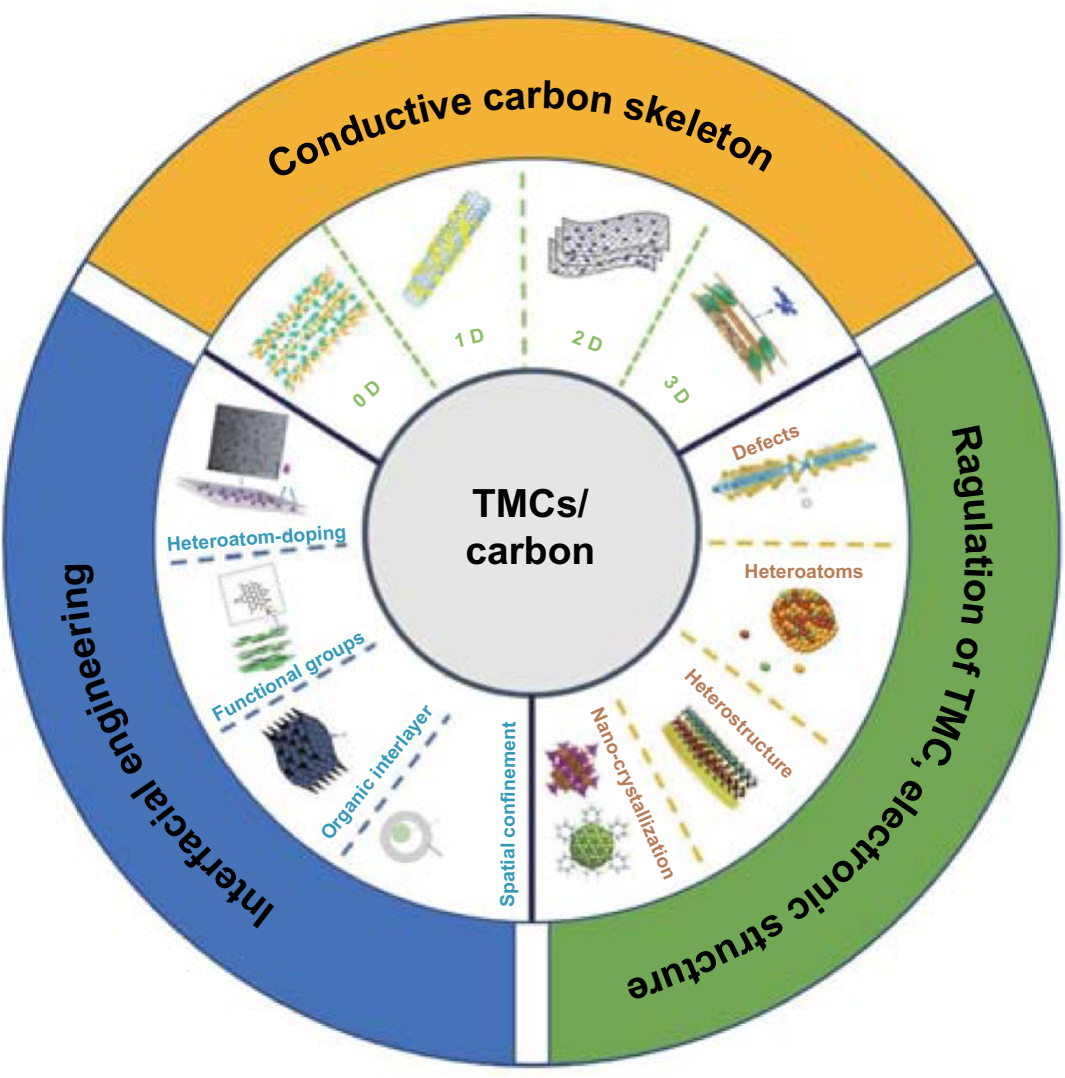
Schematic illustration of strategies of TMCs/carbon hybrid electrodes

Though TMCs/carbon composites have drawn tremendous attention for supercapacitors, the deeper research about the energy storage process and attenuation mechanisms has not yet been clearly understood. In addition, the systematic discussion about the relationship between structural design strategies, high power/energy densities, and fast pseudocapacitive reaction kinetics is critical for the practical and large-scale application of TMCs/carbon hybrid electrodes. In this review, we mainly focus on the latest advances in the field of TMCs/carbon hybrid electrodes for supercapacitors, including the construction of conductive carbon skeletons, modification of TMCs/carbon interface engineering, and regulation of electronic structures of TMCs (Fig. 1). More importantly, we propose the challenges and perspectives of developing such TMCs/carbon composites, along with the possible strategies and research directions in the future.

## Conductive Carbon Skeletons

**Fig. 2:**
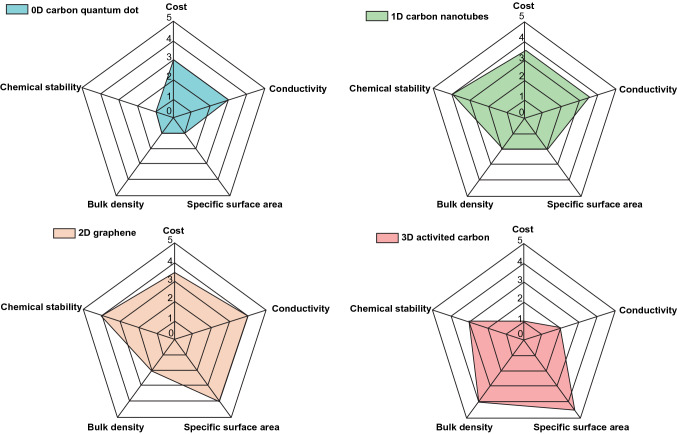
Comparison of the physicochemical properties of different dimensions carbon

The high power densities of TMC-based electrodes are commonly restricted due to their poor intrinsic conductivity and sluggish redox reaction kinetics. To solve these challenges, TMCs are usually composited with carbon skeletons, which can not only construct effective electrons/ions transfer channels, but also sustain the structural stability of the entire electrode. In recent years, carbon skeletons with various dimensions (0, 1, 2, or 3 D) have been successfully designed and introduced into the TMCs composite [23, 26–28] (Fig. 2). In this section, we will discuss the application of carbon materials as conductive skeletons in designing and fabricating TMCs/carbon hybrid electrodes from the aspect of carbon structures with different dimensions. Table 1 summarizes the electrochemical performance of TMCs/carbon hybrid electrodes for supercapacitors in recent years.

**Table 1:** Different dimensional carbon based electrode materials

Electrode material	Conductive skeleton	Electrolyte	Specific/volumetric capacity	Theoretical capacity of TMCs	Rate performance	Cycling life	References
NiCo_2_O_4_/CQDs	CQDs (0D)	2 M KOH	856 F g^−1^ at 1 A g^−1^	> 3000 F g^−1^	520 F g^−1^ at 100 A g^−1^	98.75% after 10,000 cycles at 5 A g^−1^	[29]
MnO_2_/CQDs	CQDs (0D)	1 M Na_2_SO_4_	340 F g^−1^ at 1 A g^−1^	1100 F g^−1^	260 F g^−1^ at 20 A g^−1^	80.1% after 10,000 cycles at 1 A g^−1^	[30]
Ni(OH)_2_/CQDs	CQDs (0D)	2 M KOH	2900 F g^−1^ at 1 A g^−1^	2082 F g^−1^	2051 F g^−1^ at 10 A g^−1^	–	[31]
MnO_2_/GQDs	GQDs (0D)	1 M Na_2_SO_4_	1170 F g^−1^ at 5 mV s^−1^	1100 F g^−1^	–	92.7% after10,000 cycles	[32]
V_2_O_5_/GQDs	GQDs (0D)	0.1 M H_2_SO_4_	572 F g^−1^ at 1 A g^−1^	2122 F g^−1^	346 F g^−1^ at 20 A g^−1^	92% after10,000 cycles	[33]
MnO_2_/CNT	CNT (1D)	0.5 M Na_2_SO_4_	410 F g^−1^ at 0.05 mV s^−1^	1370 F g^−1^	–	–	[34]
Ni(OH)_2_/CNT	CNT(1D)	6 M KOH	3100 F g^−1^ at 2.5 mA cm^−2^	2082 F g^−1^	2077 F g^−1^ at 50 mA cm^−2^	–	[35]
NiO/PCNF	PCNF (1D)	6 M KOH	850 F g^−1^ at 1 A g^−1^	2584 F g^−1^	748 F g^−1^ at 10 A g^−1^	96.7% after 10,000 cycles	[36]
N-GNTs@NSNs	N-GNTs (1D)	3 M KOH	2160 F g^−1^ at 6 A g^−1^	703 mAh g^−1^	1650 F g^−1^ at 40 A g^−1^	95.8% after 12,000 cycles	[37]
Ni(OH)_2_/graphene	graphene (2D)	6 M KOH	1735 F g^−1^ at 1 mV s^−1^	2082 F g^−1^	523 F g^−1^ at 50 mV s^−1^	–	[38]
Fe_2_O_3_/rGO	rGO (2D)	3 M KOH	178.3 F cm^−3^ at 1 mV s^−1^	3625 F g^−1^	123.7 F cm^−3^ at 50 mV s^−1^	83.1% after 10,000 cycles	[39]
Mn_3_O_4_/rGO	rGO (2D)	1 M Na_2_SO_4_	109 F cm^−3^ at 0.2 A cm^−3^	~ 1400 F g^−1^	20 F cm^−3^ at 20 A cm^−3^	100% after 10,000 cycles	[40]
Graphene/Co_3_O_4_	Graphene (2D)	6 M KOH	570 F g^−1^ at 1 A g^−1^	3560 F g^−1^	530 F g^−1^ at 20 A g^−1^	93% after 5000 cycles at 10 A g^−1^	[41]
3D rGO/Co_3_O_4_	3D rGO (3D)	6 M KOH	1765 F g^−1^ at 1 A g^−1^	3560 F g^−1^	1266 F g^−1^ at 20 A g^−1^	93% after 5000 cycles at 10 A g^−1^	[42]
VN/C	C (3D)	6 M KOH	392.0 F g^−1^ at 0.5 A g^−1^	1340 F g^−1^	197.9 F g^−1^ at 30 A g^−1^	82.9% after 8000 cycles	[43]
3DPC/Co_3_O_4_	3DPC (3D)	3 M KOH	423 F g^−1^ at 1 A g^−1^	3560 F g^−1^	362.5 F g^−1^ at 10A g^−1^	83% after 2000 cycles at 3 A g^−1^	[44]
CoZnNiS/CNTs/rGO	CNTs/rGO (3D)	6 M KOH	1727.0 F cm^−3^ at 1 A g^−1^	–	972.8 F cm^−3^ at 10 A g^−1^	90.6% after 10,000 cycles	[45]

### 0D Carbons (Carbon or Graphene Quantum Dots)

**Fig. 3:**
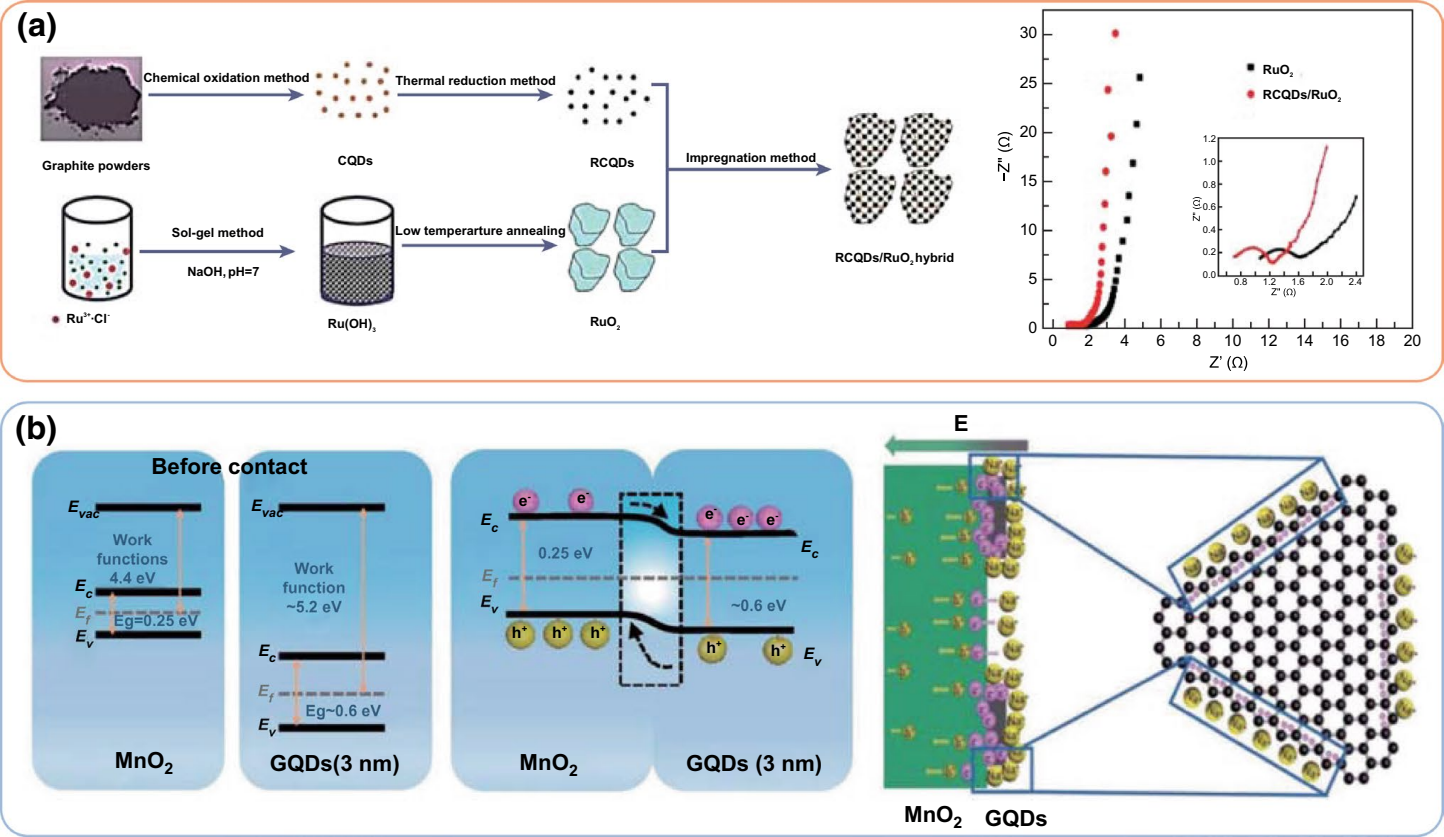
**a** Schematic illustration of preparation procedure of the RCQDs/RuO_2_ hybrid and comparison of Nyquist impedance plots of RuO_2_ and RCQDs/RuO_2_. Adapted with permission from Ref. [48] Copyright 2013 Royal Society of Chemistry. **b** Energy diagram of MnO_2_ and GQDs (≈ 3 nm) before contact and after the formation of a heterojunction, and the schematic diagram of free electrons accumulating near the GQDs surface. Adapted with permission from Ref. [32] Copyright 2018 Wiley–VCH

0D carbon materials, such as carbon quantum dots (CQDs) or graphene quantum dots (GQDs), have been diffusely studied in supercapacitors due to their small size effect, quantum tunneling effect, coulomb blockade effect, and surface effect, endowing such carbons with high electrons mobility, huge specific surface area, etc. [26, 46, 47]. In this case, TMCs combined with 0D carbon materials have emerged as promising materials for supercapacitors. For example, the reduced carbon quantum dots (RCQDs) were successfully embedded into the RuO_2_ nanoparticles (RCQDs/RuO_2_) through a facile impregnation method (As shown in Fig. 3a) [48]. The hybrid network constructed by RCQDs/RuO_2_ can provide rapid electronic transport and ionic migration paths. The RCQDs incorporated into the integral structure can effectively prevent the further agglomeration of RuO_2_ nanoparticles and vastly facilitate the fast ions/electrons transfer during charge–discharge processes (Fig. 3a), and keep the structural stability. As a result, the RCQDs/RuO_2_ electrode exhibited high specific capacitance (594 F g^−1^) and superior rate capability (77.4% capacity retention at 50 A g^−1^ compared to that at 1 A g^−1^). Moreover, the capacitance of RCQDs/RuO_2_ remained 96.9% after 5000 cycles at 5 A g^−1^, which was much higher than the RuO_2_ electrode (the rate capability and capacity retention of corresponding was only 58.3% and 73.9%, respectively).

GQDs were also introduced into the MnO_2_ nanoparticles (GQDs/MnO_2_) through the hydrothermal and plasma-enhanced chemical vapor deposition process [32]. By controlling the deposition time, a series of GQDs/MnO_2_ composites were obtained, and the GQDs (the size of 2–3 nm) were well dispersed on the surfaces of MnO_2_ sheets. Benefiting from the high conductivity and rich active sites of GQDs (Fig. 3b), the maximum specific capacitance of GQDs/MnO_2_-3 (3 represents the mass ratio of MnO_2_/GQDs) can reach to 1170 F g^−1^ at 5 mV s^−1^ and excellent cycle performance can be achieved (92.7% retention after 10,000 cycles). More importantly, as demonstrated in asymmetric supercapacitors (ASCs) using GQDs/MnO_2_-3 and nitrogen-doped graphene as anode and cathode, respectively, the superior energy density of 79 Wh kg^−1^ at an ultrahigh power density of 12,351 W kg^−1^ can be obtained in an aqueous electrolyte with ultrawide voltage window (2.3 V). In addition, the GQDs embed to V_2_O_5_ interlayer, constructing VNS-GQDs positive electrode composite material was recently reported by Ganganboina and co-workers via a solvothermal treatment process [33]. Due to the high conductivity of graphene quantum dots, the VNS-GQDs exhibited an ultra-high specific capacitance of 572 F g^−1^ at 1 A g^−1^, which could keep 92% retention after 10,000 charge–discharge cycles, much higher than that of VNS (227 F g^−1^). Due to the synergistic effect of GQDs and VNS nanosheets, the assembled asymmetric device (VNS-GQDs//MCS) demonstrated a superior energy density of 20.62 Wh kg^−1^ at the superhigh power density of 14.86 kW kg^−1^.

In addition to the metal oxides, other transition metal derivatives, such as metal hydroxides/sulfides, can also be decorated by GQDs. For example, the CQDs/Ni(OH)_2_ with a lamellar structure was prepared by Wei and co-workers through a facile hydrothermal method [31]. The introduction of CQDs greatly improved the rate performance and specific capacitance of the overall electrode. As a result, the CQDs/Ni(OH)_2_ electrode exhibited a high specific capacitance of 2900 F g^−1^ at 1 A g^−1^, and the capacitance still remained at 2051 F g^−1^ even at a high current density of 10 A g^−1^. Furthermore, Huang et al. fabricated GQDs/NiCo_2_S_4_ composite that GQDs decorated hierarchical-like hollow NiCo_2_S_4_ nanostructures by simple two-step hydrothermal reactions [49]. The introduction of the GQDs can change the surface morphology and internal structure of NiCo_2_S_4_ nanowires, indicating the unique nanostructures result in low impedance, large specific surface area, and more redox active sites, more interspaces and pathways for ion diffusion. As a result, the prepared GQDs/NiCo_2_S_4_ electrode exhibited high specific capacitance (678.22 F g^−1^ at 0.2 A g^−1^) and superior cycling stability (94% retention after 5000 cycles).

The greatly improved electrochemical performance of the 0D carbons decorated TMCs electrode could be attributed to two reasons: (1) the CQDs or GQDs incorporated in the composite can provide additional capacitance and construct highly conductive networks inside the micro-nano-scaled regions, which promotes the rapid charge transport and ionic diffusion, boosts the fast redox reactions, ensuring the excellent rate performance; (2) a small number of oxygen-containing functional groups (mainly carboxyl) at the edges of CQDs or GQDs can form strong and stable chemical interactions between the 0D carbon and TMCs through the formation of C–O–M covalent bonds (M represents metal atoms), which is beneficial for improving the rate and cycling performance. However, though the 0D carbons generally have been used to enhance some pseudocapacitive materials conductivity, the relative lower specific capacitance and easy agglomeration of such carbons still need to be solved.

### 1D Carbons (Nanotubes or Nanofibers)

Owing to the high conductivity, good flexibility, and superior functionality, 1D carbon materials, such as carbon nanofibers (CNF) and carbon nanotubes (CNTs), have been widely used as individual electrode materials or conductive substrates for supercapacitors [50, 51]. In the process, the TMCs are commonly in situ introduced into the surface of 1D CNTs through physical or chemical methods. Such a strategy can not only effectively control the morphology of TMCs, but also greatly improve the overall electrical conductivity of 1D TMCs/carbon composites, which is beneficial for the improvement of both power and energy densities. For example, the Ni(OH)_2_ could be irreversibly transformed into NiO_x_ during the energy storage process and commonly resulted in the poor rate capability and cycle stability of such hydroxide. To address the issue, Tang et al. [35] have prepared the Ni(OH)_2_/CNTs/NF electrode through the CVD and chemical bath deposition (CBD) process. After the introduction of high conductivity CNTs, the intrinsic drawbacks of Ni(OH)_2_ could be effectively exempted. The electrochemical testing showed that the Ni(OH)_2_/CNTs/NF electrode exhibited both high rate capacity (3300 F g^−1^ at 0.5 A g^−1^, 2211 F g^−1^ at 10 A g^−1^) and excellent cycling stability (87% retention after 3000 cycles). In addition, when assembled as asymmetric supercapacitors using Ni(OH)_2_/CNTs/NF and AC as anode and cathode, respectively, the device indicated an ultra-high energy density of 32.5 Wh kg^−1^ at a power density of 1800 W kg^−1^. Such an obvious improvement can be attributed to the elaborate design of a composite structure, in which the active Ni(OH)_2_ nanoparticles are effectively anchored on the surface of highly conductive CNTs/NF current collectors and dramatically impeded the phase transformation of Ni(OH)_2_.

**Fig. 4:**
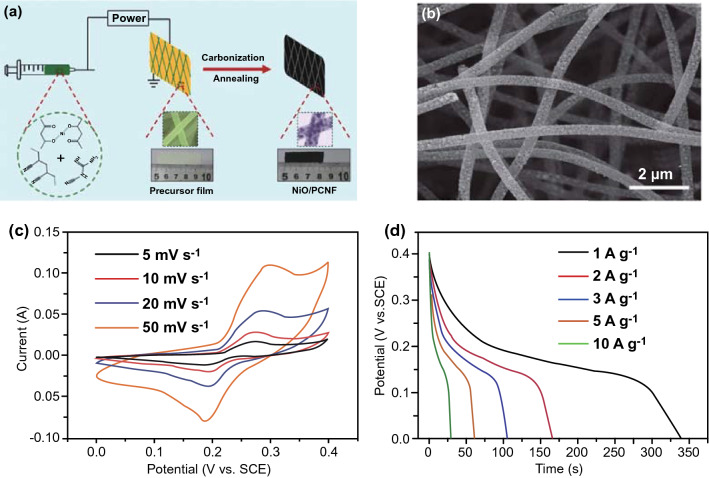
**a** Schematic illustration of the synthesis of free-standing NiO/PCNF composite film. **b**, **c** and **d** SEM images, CV curves, and discharge voltage profiles of NiO/PCNF-0.75. Adapted with permission from Ref. [36] Copyright 2018 Wiley–VCH

Besides the 1D carbon skeleton surface, the TMCs can also be confined into the cavities of carbon nanofibers [52]. The interspace provided by the 1D carbon skeleton can effectively prevent the aggregation and phase transformation of TMC-based electrodes. For example, the P-doped Co_3_O_4_ ultrafine nanoparticles have been successfully encapsulated into P, N co-doped carbon nanowires (P-Co_3_O_4_@P, N–C) by a pyrolysis-oxidation-phosphorization method [53]. Because of the strong fastened effect of electroactive Co_3_O_4_ into the conductive carbon substrate, the agglomeration of active materials was prevented. Moreover, the good keeping of 1D oriented arrangement hybrid composites can provide a huge accessible surface area and hierarchically porous characteristic, which are beneficial for the sufficient permeation and migration of electrolyte ions. When assembled with Co@P/N–C, the P-Co_3_O_4_@P/N–C//Co@P/N–C asymmetric supercapacitor could achieve an outstanding energy density of 47.6 Wh kg^−1^ at the high power density of 750 W kg^−1^, indicating the impressive supercapacitor possessing an enormous potential in the practical applications. In addition, a NiO nanoparticle-dispersed electrospun N-doped porous CNF (NiO/PCNF) free-standing film electrode was fabricated using dicyandiamide(DCDA) and PAN as CNF precursors and nickel acetylacetone as Ni sources by Li et al. (Fig. 4a) [36]. The NiO nanoparticles were uniformly dispersed in the framework of N-doped porous CNF (Fig. 4b). Such a 1D fibrous feature and the interwoven conductive skeleton are beneficial for the fast transfer of electrolyte ions/electrons through the in-plane direction. The NiO/PCNF-0.75 (0.75 represents the weight ratio of DCDA and nickel acetylacetone) showed the maximum specific capacitance of 850 F g^−1^ at 1 A g^−1^, and high specific capacitance was retained 748 F g^−1^ even at 10 A g^−1^, meaning outstanding rate capability (Fig. 4c, d).

Owing to the high electrical conductivity, superior mechanical strength, and flexible capability, such 1D TMCs/carbon composites have also been widely researched in wearable device applications [50]. For example, Hu et al. [34] developed a MnO_2_-CNT-textile electrode through the electrodeposit method of MnO_2_ onto the highly conductive CNT with polyester coatings, which exhibited a large mass loading (8.3 mg cm^−2^) and high areal capacitance (2.8 F cm^−2^) in a wearable device. The textile fibers wattled by CNT with high conductivity could largely promote the electrolyte ions/electrons transfer through the in-plane direction, and the MnO_2_-CNT-textile electrode exhibited a high energy density of 5–20 Wh kg^−1^ at the high power density of 13 kW kg^−1^. In another article, Salman and co-workers successfully integrated tungsten nitride (WN) with reduced graphene oxide fibers (rGOF) to obtain a WN-rGOF hybrid by a simple hydrothermal method [54]. Owing to the high tensile strength, excellent flexibility and high electrical conductivity of rGOF, large capacitance and high chemical stability of WN electrode can be obtained. The energy density of WN-rGOF-based hybrid supercapacitors showed a great energy density of 1.448 mWh cm^−3^. More importantly, the WN-rGOF electrode showed high capacitance retention of 84.7% after 10,000 cycles even under severe mechanical deformation conditions, exhibiting a fascinating advantage for wearable device applications in the future.

1D carbons showed great potential for supercapacitors application, especially flexible wearable devices. However, higher demands have been made for flexible energy-storage systems along with the rapid development of the society, which needs to improve the areal/volumetric performances of 1D carbon. Notably, the form of free-standing TMCs/carbon films attracted more and more attention owing to no additional binders, conductors, or collectors. Compared with TMCs/carbon powders, it can increase the mass loading of active materials, simplify the electrode preparation process, and avoid uncontrollable side reactions, but it needs more advanced technologies. Therefore, the development and application of free-standing film electrodes may be an important development direction of the energy storage industry.

### 2D Carbons

**Fig. 5:**
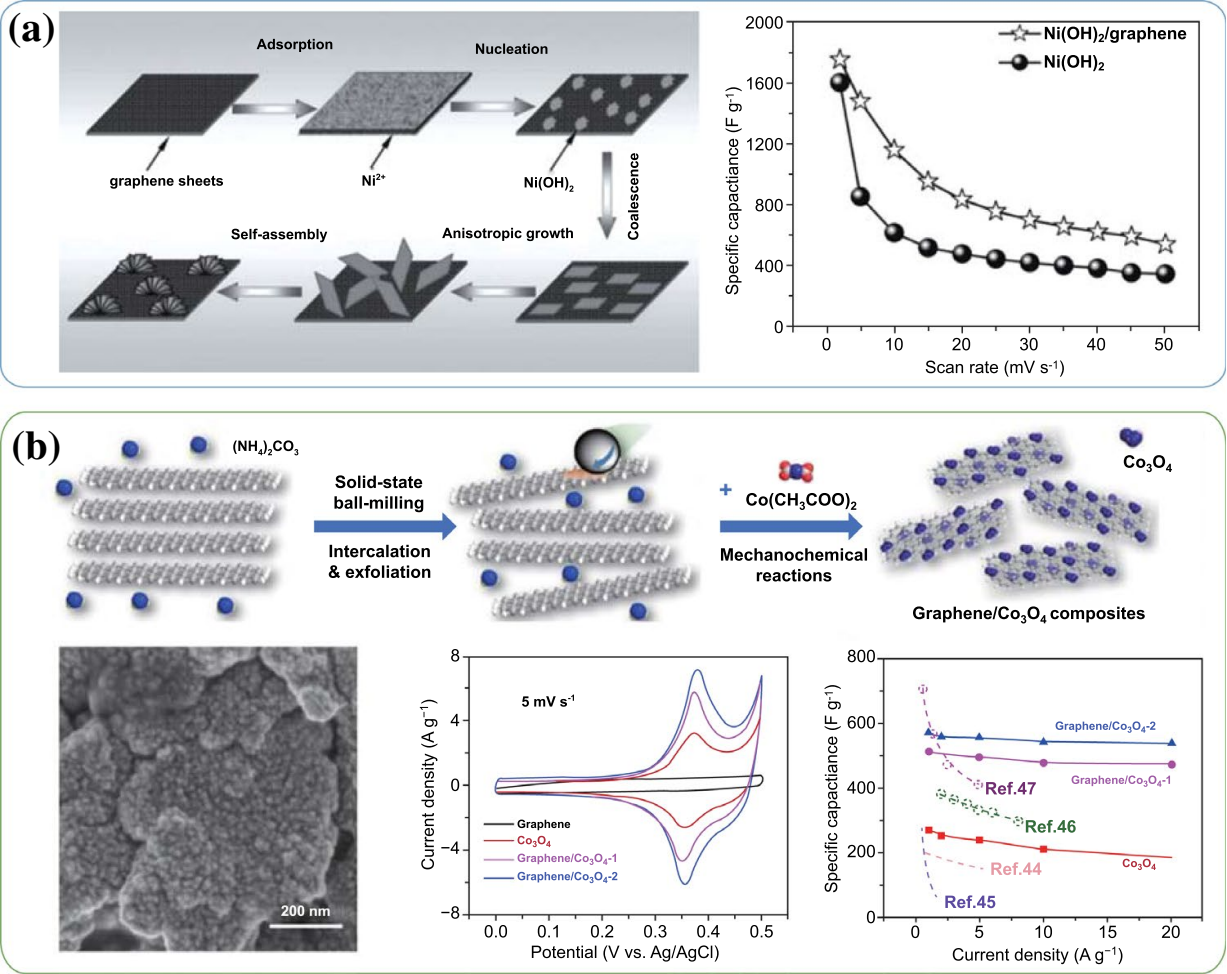
**a** Schematic illustration for the possible formation of the Ni(OH)_2_/graphene and the specific capacitances of Ni(OH)_2_/graphene and Ni(OH)_2_ at different scan rates. Adapted with permission from Ref. [38] Copyright 2012 Wiley–VCH. **b** Schematic illustration for the preparation of graphene/Co_3_O_4_ composites, SEM images of graphene/Co_3_O_4_-2, CV curves at 5 mV s^−1^ of graphene, Co_3_O_4_, graphene/Co_3_O_4_-1 and graphene/Co_3_O_4_-2, and their specific capacitances at various current densities. Adapted with permission from Ref. [41] Copyright 2020 Elsevier

Due to the ultra-high specific surface area (2630 m^2^ g^−1^) and excellent conductivity, studies about the 2D graphene-based materials applied in energy storage and conversion have sprung up since its first report in 2004 by Novoselov and Geim [55, 56]. More specially, graphene also has been widely studied as a conductive substrate and flexible host in the synthesis of graphene/TMCs composites [15, 57, 58]. The continuously conjugated *sp*^2^-C skeleton of graphene can provide efficient ions/electrons transfer channels, which is not only beneficial for the improvement of rate performance of TMCs, but also significant for the long charge/discharge cycling stability. For example, our group previously prepared Ni(OH)_2_/graphene hybrid material via the microwave heating method [38]. Structural characterization demonstrated that the as-prepared Ni(OH)_2_ with hierarchical flowerlike morphology were homogeneously decorated on the graphene nanosheets. Compared with pure Ni(OH)_2_, the hybrid material showed higher specific capacitance and more superior rate capability (As shown in Fig. 5a). Moreover, the Ni(OH)_2_/graphene//porous graphene-based asymmetric supercapacitor exhibited a high energy density of 77.8 Wh kg^−1^ at 174.7 W kg^−1^, which could still retain 13.5 Wh kg^−1^ at an ultrahigh power density of 15.2 kW kg^−1^. The superior performance mainly attributes to high conductivity and specific surface area of graphene-based conductive skeleton and high theoretical specific capacitance of Ni(OH)_2_.

A class of hybrid Fe_2_O_3_ nanoclusters/rGO papers (Fe_2_O_3_/ rGO)-based negative electrodes were also prepared by Hu and co-workers through a facile hydrothermal method and followed by an electrochemical reduction strategy [39]. When tested in 3 M KOH aqueous electrolyte, the hybrid Fe_2_O_3_/rGO electrode obtained both high volumetric energy and power density (0.056 Wh cm^−3^ and 6.21 W cm^−3^). Such excellent electrochemical performance can be attributed to the conductive skeleton and flexible host of rGO nanosheets, and the high pseudocapacitance provided by Fe_2_O_3_ nanoparticles. As shown in Fig. 5b, Jiang et al. developed a simple, productive, and economical one-pot ball-milling manner to produce a series of graphene/Co_3_O_4_ composites [41]. SEM image indicated that the Co_3_O_4_ nanoparticles were uniformly anchored on the graphene surface. Graphene as a conductive skeleton can offer fast ion transport pathway. Compared with other composites, the as-prepared graphene/Co_3_O_4_-2 (2 represents the graphene content) displayed the most distinguished specific capacitance of 530 F g^−1^ at 20 A g^−1^ (Fig. 5b). When assembled as asymmetric supercapacitors using graphene as anode and graphene/Co_3_O_4_-2 as a cathode, the energy density of such ASCs could reach 67.5 W h kg^−1^ at the high power density of 0.8 kW kg^−1^.

Though the graphene in the construction of TMC-based composite electrodes has been widely employed, the serious agglomeration and restacking issues due to the interplanar *π*–*π* interaction and Van der Waals forces always impede its practical popularization, which is urgent to be solved [59]. Moreover, graphene have outstanding electronic conductivity on the horizontal plane; however, its vertical conductivity is low and usually does not achieve ion cross-layer transmission. To address these problems, some methods have been developed, such as pores-creation using KOH/H_2_O_2_ treatment, lead-in spacer, and design of ribbons [60, 61].

### 3D Carbons

**Fig. 6:**
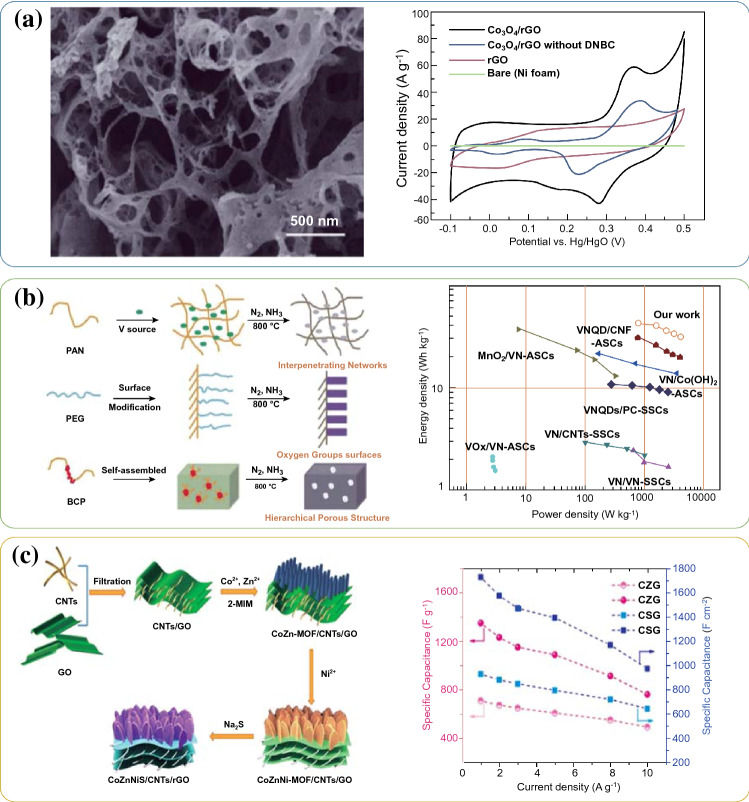
**a** SEM image of the 3D graphene frameworks/Co_3_O_4_ composites and CV curves at 50 mV s^−1^ of the various sample. Adapted with permission from Ref. [42] Copyright 2017 Wiley–VCH. **b** Schematic representation of the fabrication strategy and Ragone plots of the VN/C. Adapted with permission from Ref. [43] Copyright 2018 Springer. **c** Schematic illustration of the preparation of the CoZnNiS/CNTs/rGO composite and their volumetric and gravimetric capacitance at different current densities. Adapted with permission from Ref. [45] Copyright 2020 Elsevier

To address the above aggregation issue of graphene, reasonably constructing 3D carbon frameworks, such as 3D porous carbon, 3D graphene building, or 3D cross-linked carbon network, etc., as highly conductive substrates have been raised [62–68]. These carbon skeletons with unique 3D morphology usually exhibit hierarchical porous structure and ultra-high surface area, which can provide abundant electrolyte ions/electrons transfer channels and sufficient growing space for TMCs. In this context, Bao et al. prepared 3D graphene frameworks/Co_3_O_4_ composites via a thermal explosion method [42]. As depicted in Fig. 6a, 3D graphene frameworks with macropores acting as the conductive skeleton can facilitate the rapid transfer of electrolyte ions/electrons. In addition, the Co_3_O_4_ nanoparticles with about 4.6–9.4 nm diameters were uniformly and densely distributed on the 3D carbon frameworks. When 3D graphene/Co_3_O_4_ composites were used as electrode material for supercapacitors, high specific capacitance (≈ 1765 F g^−1^ at a current density of 1 A g^−1^) could be observed, and retention kept 93% even after 5000 cycles at a highly constant current density of 10 A g^−1^. Moreover, Xu et al. successfully prepared a hybrid material that VN nanoparticles incorporated into a 3D carbon matrix (3D VN/C) through the multi-phase polymeric strategy (Fig. 6b) [43]. The VN nanoparticles are uniformly distributed among the 3D carbon networks, in which the carbon matrix can not only serve as a 3D conductive network but also prevent the VN aggregation. As a result, the 3D VN/C membranes electrode showed excellent electrochemical performance, including high energy density (43.0 Wh kg^−1^ at a power density of 800 W kg^−1^) and good cycling stability (82.9% at 1.0 A g^−1^ after 8000 cycles). More importantly, when the power density reached 4 kW kg^−1^, the device still performed a high energy density of 32.3 Wh kg^−1^.

Apart from the usage of single carbon to build 3D conductive carbon skeletons, the substrates can also be constructed by two or more kinds of carbon units. For example, Qing et al. [69] embedded highly crystallized GQDs onto activated carbons (AC) to construct 3D overall conductive networks, which could greatly improve the capacity and rate performances in contrast to pure ACs. The small-sized GQDs are more beneficial to establish evenly distributed conductive networks, which can accelerate the electron/ion transport kinetics in carbon frameworks. Liu et al. [45] designed and prepared MOFs-derived ZnCoNiS nanosheet arrays based on a 3D rGO/CNTs conductive substrate (CoZnNiS/CNTs/rGO) (As depicted in Fig. 6c). The 3D rGO/CNTs network endowed high conductivity of the whole electrode and greatly promoted the electrons transfer kinetics while shortened the electrolyte ions diffusion paths. Moreover, the rough surface of CoZnNiS nanosheets could provide abundant exposing surfaces and active sites, enhancing the redox reaction rate. As a result, the composite film electrode displayed a maximum specific capacitance and high volumetric specific capacitance (1349.2 F g^−1^ and 1727.0 F cm^−3^ at 1 A g^−1^, respectively). When used CoZnNiS/CNTs/rGO and carbon sphere/rGO electrode to assemble hybrid supercapacitor, the device showed a superior energy density of 60.4 Wh kg^−1^ at an ultrahigh power density of 1200 W kg^−1^.

To sum up, regardless of the 0D, 1D, 2D, or 3D carbon skeletons, the reasonable design of carbon structures with high conductivity and large specific surface areas to satisfy the high mass loading of TMCs is quietly important. Moreover, how to design ideal carbon skeletons, which exhibit both high bulk densities and porous structure, seems a big challenge. Particularly, the relationships between the holistic devices’ energy density and the ratio of TMCs to carbon-based host have not been reported so far, the content of porous carbon host affects the conductivity and capacitance of the whole electrode that the content of porous carbon host too high to achieve high volume density leading higher electrolyte consumption, too low causing of high charge resistance. In the practical application of TMCs, the fast reaction kinetics of the pseudocapacitive energy storage process commonly need the synergistic effect of conductivity, ions transfer channels, interface stability, etc. Therefore, the systematic design of carbon skeletons for the TMCs/carbon based supercapacitors with high energy/power densities may be the research direction in the future.

## Interface Engineering

Besides the construction of conductive carbon skeletons, the well-designed interface engineering between carbon networks and TMCs is another indispensable role for TMC-based electrodes which exhibit improved energy density and cycle life [70]. Due to the nano-size effect, TMCs nanoparticles with high surface energy are easy to aggregate during the energy storage process, which can directly lead to the capacity fading, resulting in the hindrance of scaled application of TMCs/carbon-based supercapacitors [71, 72]. Moreover, the roughly physical combination of TMCs and carbon skeletons usually causes high interface impedance and cuts off the electron transfer channels across the heterogeneous interfaces. Therefore, the ideal design of carbon/TMCs interfaces is vitally important for the further enhancement of electrochemical performance. In this section, we will summarize the recent progress of the interface engineering between carbon and TMCs from the aspects of heteroatom-doping, functional group modification, organic interlayer, and spatial confinement (Table 2).

**Table 2:** Comparison of different interfacial strategies

Types of strategies	TMCs/carbon	Synthetic methods	Rate ability	References
Heteroatom-doped carbon skeletons	Fe_2_O_3_ ND@NG	Solvothermal	201 at 5 A g^−1^, 140 at 50 A g^−1^	[73]
	Ni/Co-OOH/NSCF	Electrochemical activation	853 C g^−1^ at 5 A g^−1^, 518.4 at 20 A g^−1^	[17]
Covalently heterogeneous interface	Ni(OH)_2_/af-GQDs	Impregnation	2653 F g^−1^ at 1 A g^−1^, 1658 at 20 A g^−1^	[96]
	Mn_3_O_4_-rGO	Thermal treatment	561.5 C g^−1^ at 1 A g^−1^, 363.8 at 20 A g^−1^	[75]
Organic interlayer	CuO-PANI-rGO	hydrothermal	634.4 F g^−1^ at 0.5 A g^−1^, 513.2 at 20 A g^−1^	[76]
	ACNF/PANI/NiO	Carbonization	1157 F g^−1^ at 1 A g^−1^, 720 at 10 A g^−1^	[77]
Spatial confinement	VNNDs/CNSs	Carbonization	573 F g^−1^ at 0.5 A g^−1^, 334.8 at 100 A g^−1^	[78]
	NiS–NC HS	Hydrothermal	1170 F g^−1^ at 0.5 A g^−1^, 843.5 at 10 A g^−1^	[79]

### Heteroatom-Doped Carbon Skeletons

**Fig. 7:**
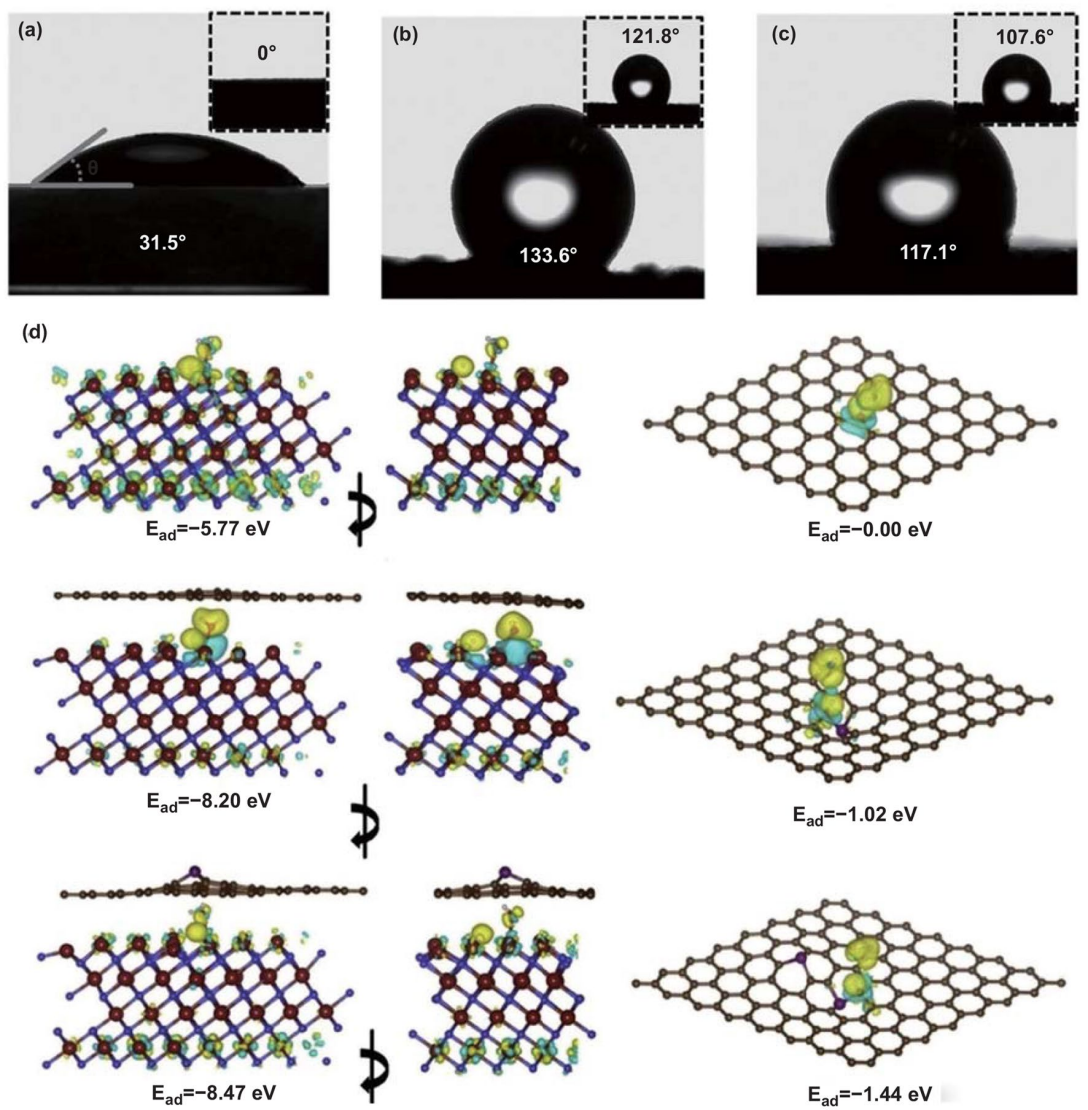
**a**–**c** Comparison of water contact angles of N-carbon foam, carbon foam and carbon cloth. Adapted with permission from Ref [84]. Copyright 2016 Wiley–VCH. **d** DFT simulations of binding energy between MoN and P-doped carbon. Adapted with permission from Ref. [85] Copyright 2019 Elsevier

**Fig. 8:**
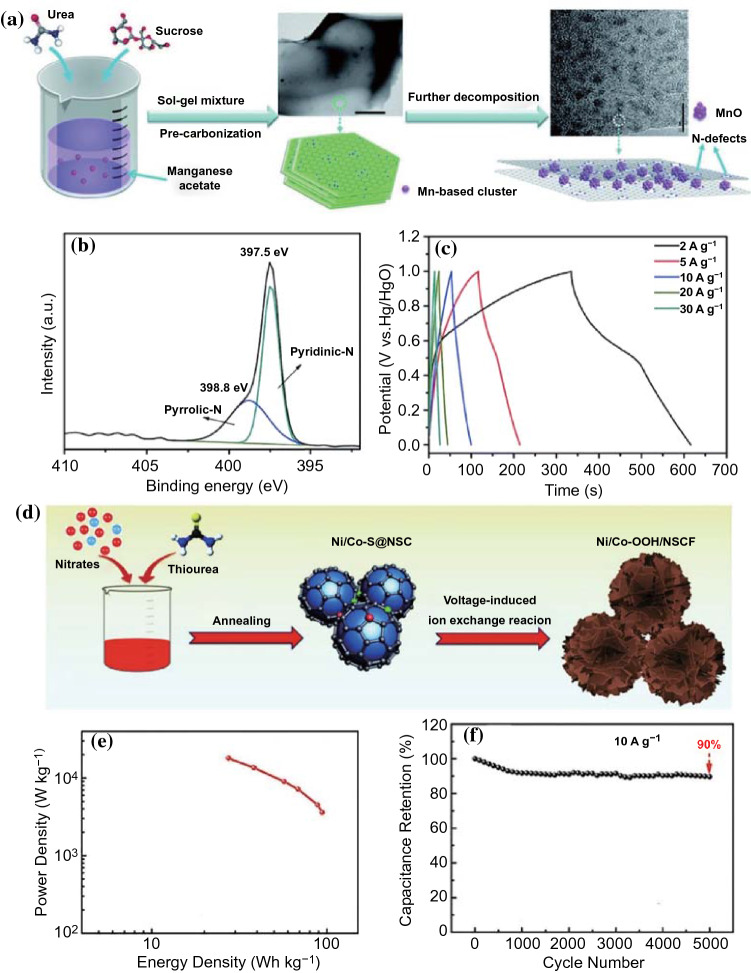
**a** Synthetic procedure picture of MnO@NCs. **b** High-resolution XPS of N 1* s* peak of MnO@NCs. **c** Galvanostatic charge–discharge curves MnO@NCs of concerning current densities. Adapted with permission from Ref. [86] Copyright 2014 Royal Society of Chemistry. **d**–**f** Schematic illustration for the synthesis and Ragone plots, cycling performance of Ni/Co–OOH/NSCF. Adapted with permission from Ref. [17] Copyright 2020 Elsevier

Heteroatoms doping (such as B, N, P, and S) of carbon skeleton is an effective way to construct strong TMCs–carbon interfaces [80]. Density functional theory (DFT) calculations verified that the density of states and charge population of carbon materials have been dramatically altered after the heteroatoms doping [81]. Previous works demonstrated that the electrochemical conductivity and wettability of carbon materials were remarkably improved by the heteroatoms doping, which was beneficial for the enhancement of the electrochemical activity of TMCs/carbon-based electrodes (Fig. 7a–c) [82–84]. For example, Dubal and Abdel-Azeim et al. [85] confirmed that the introduction of heteroatoms in carbon matrix could enhance the binding energy between TMCs and carbon (Fig. 7d). In this case, Yang et al. [86] successfully using urea as N source manufactured ultrasmall MnO nanoparticles inserted into N-rich carbon nanosheets (MnO@NCs) hybrid by a facile tactic (Fig. 8a). As shown in Fig. 8b, XPS confirmed that N atoms were successfully introduced into the carbon skeleton, and the ultrasmall MnO nanoparticles (around 2–4 nm) were uniformly anchored on the N-rich carbon nanosheets. Moreover, the copious nitrogen species doped in the carbon skeletons altered the local electron distribution, and the nanoparticles were preferentially in situ nucleated at the N-rich sites, which was beneficial for the uniform dispersion of MnO nanoparticles and offered powerful support for the large mass loading of MnO nanoparticles. Consequently, as assembled in an asymmetric device, the MnO@NCs//AC demonstrated a striking energy density of 21.6 Wh kg^−1^ at a high power density of 12.4 kW kg^−1^.

In another report, the composite electrode of Fe_2_O_3_ nanodots supported by nitrogen-doped graphene sheets (Fe_2_O_3_ ND@NG) was prepared through a facile one-pot method [73]. At a current density of 1 A g^−1^, the Fe_2_O_3_ ND@NG-0.75 (0.75 represents mass ratios of precursor) with the highest N content electrode exhibited the highest specific capacitance of 274 F g^−1^, which could still arrive to 140 F g^−1^ even at a high current density of 50 A g^−1^, indicating a superior rate performance. The excellent electrochemical performance may be attributed to that the nitrogen doping in graphene not only endows graphene with a high charge mobility rate and extra capacitance, but also increases the binding energy between Fe_2_O_3_ NDs and NG to build a stable heterogeneous interface. In addition, Miao et al. selected MOFs as precursors to obtain a series of CoSe_2_/NC hybrid materials with abundant N content [87]. N doping not only enhanced the electric conductivity and wettability of carbon skeletons, but also contributed additional pseudocapacitance. Compared with other hybrids, the CoSe_2_/NC-400 electrode presented superior electrochemical performance, including high capacity (120.2 mAh g^−1^ at 1 A g^−1^), good rate capability (61.2% from 1 to 20 A g^−1^), and remarkable cyclic ability (retaining 92% after 10,000 cycles). Furthermore, the CoSe_2_/NC-400//AC-based asymmetric device showed an outstanding energy density of 40.9 Wh kg^−1^ at a high power density of 980 W kg^−1^.

Besides single atom doping, co-doping of multiple atoms is also widely investigated [82]. As reported, various heteroatoms co-dopped in the carbon matrix could play specific roles, such as N atom boosting capacitance [88], P atom operating potential window in aqueous electrolyte [89, 90], O atom promoting the electrode–electrolyte interaction [91, 92], and so on [93]. Moreover, the multiple atoms co-doped in the carbon skeleton can play a synergistic coupling effect and effectively improve the electrochemical activities of TMCs/carbon-based electrodes [94]. For example, Hou et al. [17] synthesized Ni/Co-OOH nanosheets decorated with N, S co-doped carbon fragments (NSCF) through an in situ electrochemical self-reconstruction method (Fig. 8d). Even at a high mass loading, the Ni/Co-OOH/NSCF electrode still delivered an ultrahigh capacity and excellent rate capability. Furthermore, as depicted in Fig. 8e, f, an outstanding energy density of 94.3 Wh kg^−1^ at an ultrahigh power density of 3.6 kW kg^−1^ could be observed based on the assembled asymmetric supercapacitor of Ni/Co-OOH/NSCF//Fe@N-doped carbon. In another article, Zhao and co-workers successfully fabricated MoS_2_/nitrogen-phosphorus co-doped graphene composite (MoS_2_/NPG) via a one-pot hydrothermal strategy [95]. The introduction of N and P atoms could greatly enhance the conductivity and wettability of graphene. After N and P doping, the intimate contact between MoS_2_ and graphene was largely improved, which could efficiently reduce the electrolyte ions/electrons transfer impedance inside the whole electrode, even across the electrode/electrolyte interfaces. Owing to the synergistic effect of N and P doping, the MoS_2_/NPG composite showed an outstanding specific capacitance (588 F g^−1^) in 1 M Na_2_SO_4_ aqueous solution. Moreover, the MoS_2_/NPG electrode-based symmetric supercapacitor device presented a high energy density of 24.34 Wh kg^−1^ at 300 W kg^−1^. Even at an ultrahigh power density of 5994 W kg^−1^, the energy density still remained 6.66 Wh kg^−1^.

Despite the heteroatoms-doping carbon displayed improved electrochemical performance in aqueous electrolytes, it may be counterproductive in organic electrolytes. Therefore, systematic study of influence of heteroatom doping on the surface chemistry of carbon materials will be a focus of future research. Moreover, reasonable doping amount and doping type also have a great influence on the electrochemical properties of carbon materials. The amount of dopants in carbon is too low to provide more faradaic pseudocapacitances, too high will sacrifice high conductivity, and different doping types perform various functions such as the forms of nitrogen atoms doped including pyridine N, pyrrolic N, graphitic N, etc.

### Covalently Heterogeneous Interface

Be similar to the heteroatoms doping, the introduction of functional groups onto carbon skeleton surface is another necessary strategy to ensure the formation of efficient covalent grafting between TMCs and carbon skeleton through the construction of M–O/N/P/S-C bonds, in which the M represents TMCs, the O/N/P/S represents functional groups, and C represents carbon skeletons. Using strong oxidizing acids (such as nitric and sulfuric acid) to functionalize the carbon surface is the most common method, which can introduce functional groups like amino, carboxyl, hydroxyl, phosphate, or thiol [70]. These superficial functional groups can not only control the loading amount of TMCs but also increase the infiltration of electrode materials in the electrolyte. More importantly, the covalent interfaces between TMCs and carbon are beneficial for the improvement of structural stability during the long charge/discharge process.

For example, Ko et al. [74] prepared layer-by-layer assembly films consisting of amine-functionalized carbon nanotubes (CNTs) and oleic acid-stabilized transition metal oxide nanoparticles (OA-TMO NPs) via the direct construction of covalent-bonding interfaces. The covalent interfaces between CNTs and TMO NPs can reduce the internal interfacial resistance and promote fast electron/ions transfer. Therefore, the CNTs/OA-TMO NPs electrode showed excellent electrochemical performance, including high specific capacitance and excellent cycling stability (e.g., the CNTs/OA-MnO NPs performed the capacity of 305 F cm^−3^ at 5 mV s^−1^ and capacitance retention of 106% after 10,000 cycles). In another report, Ni(OH)_2_/amino-functionalized graphene quantum dots (Ni(OH)_2_/af-GQDs) composite was fabricated by Zheng and his co-workers via a facile mixture stirring method [96]. Obviously, the introduction of amino-groups on graphene surfaces could remarkably promote the ions transport and enhance anchor forces for Ni^2^^+^, which were beneficial for the improvement of electrochemical capacitance. The asymmetric supercapacitor with the Ni(OH)_2_/af-GQDs as a positive electrode and electrochemical-exfoliated graphene as negative electrode showed a high energy density of 46.5 Wh kg^−1^ at 1 kW kg^−1^, which still remained at 16.8 Wh kg^−1^ even at a high power density of 9 kW kg^−1^.

**Fig. 9:**
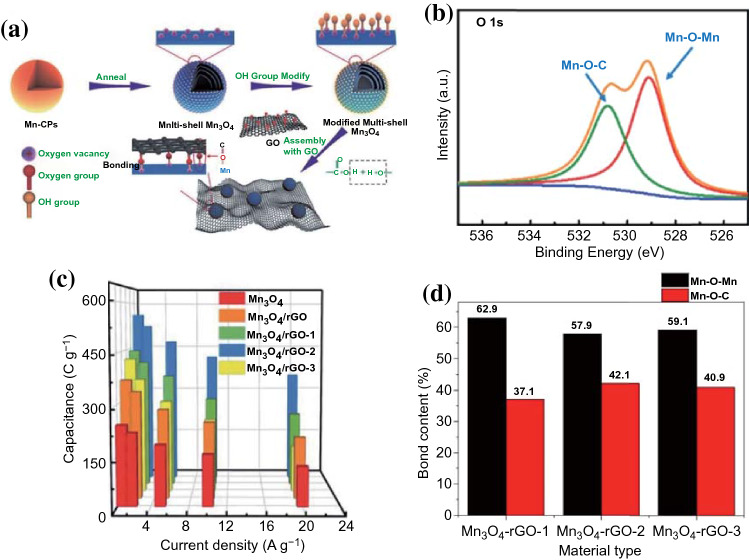
**a** Schematic illustration for the fabrication of Mn_3_O_4_-rGO. **b** O 1s core-level spectra of Mn_3_O_4_-rGO. **c** The specific capacitance of all samples at different current densities. **d** Contents of Mn–O–C and Mn–O–Mn in all Mn_3_O_4_-rGO samples. Adapted with permission from Ref. [75] Copyright 2019 Royal Society of Chemistry

In addition, Jia et al. [75] encapsulated the Mn_3_O_4_ hollow spheres with modified multi-shell numbers onto the reduced graphene oxide (rGO) surface to obtain the Mn_3_O_4_-rGO hybrid materials through oxygen vacancy-assisted hydroxyl modifying method (Fig. 9a). The rGO and multi-shelled Mn_3_O_4_ hollow spheres could form Mn-C-O bond with strong binding forces, which could be identified by the XPS O 1 s spectrum with the existence of Mn–O-C specific peak (Fig. 9b). As shown in Fig. 9c, d, the Mn_3_O_4_-rGO-2 (2 represents different rGO weights) with large Mn–O–C content (42.1%) exhibited a much higher specific capacitance (561.5 C g^−1^ at 1 A g^−1^) and more excellent cycling stability (98% retention after 10,000 cycles). In comparison to the other two hybrid materials, the improved electrochemical performance can be attributed to the construction of covalent heterogeneous interfaces which can ensure the fast charge/discharge reaction kinetics and ultra-long cycling stabilities [75]. Therefore, to ensure fast and reversible Faradaic redox reactions, the design of strong covalent grafting between carbons and TMCs is a promising strategy that reduces the charge transfer resistance at the interface.

### Organic Interlayer

In order to improve the interface stability between TMCs and carbon skeleton, the covalent introduction of organic interlayers onto carbon surface to form the ternary composite is also an efficient method. The organic interlayer between carbon skeleton and TMCs can construct a firm adhesive layer, like glue, connecting the conductive carbon skeleton and pseudocapacitive TMCs. Nowadays, many conductive polymers as carbon coatings have been widely researched, like polyaniline (PANI), polypyrrole (PPy), polythiophene (PTh), etc. [97–99].

**Fig. 10:**
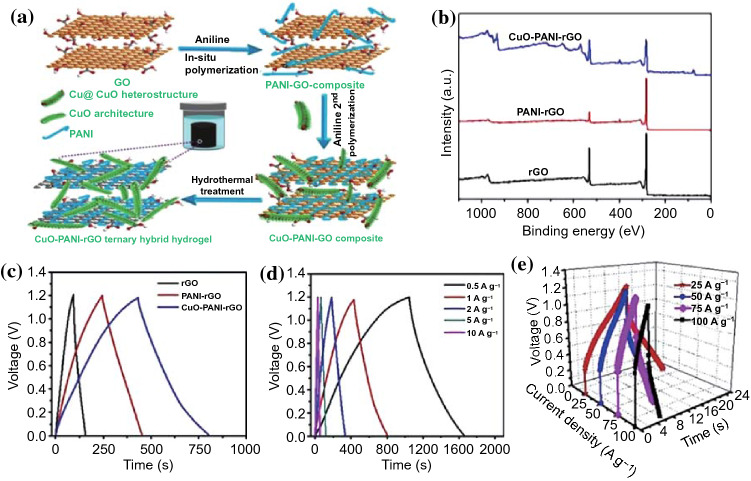
**a** Schematic illustration for the self-assembly procedure of the CuO-PANI-rGO ternary hybrid sample. **b** XPS survey spectra of the typical pure rGO sample, PANI-rGO binary hybrid sample, CuO-PANI-rGO ternary hybrid sample. **c** GCD curves of the typical CuO-PANI-rGO ternary hybrid sample compared with rGO and PANI-rGO samples at current density of 1.0 A g^−1^. **d**, **e** GCD curves of the typical CuO-PANI-rGO ternary hybrid sample at different current densities from 0.5 to 10 A g^−1^. Adapted with permission from Ref. [76] Copyright 2016 Elsevier

Taking polyaniline as an example, Zhu et al. [76] fabricated CuO-PANI-rGO ternary hybrid via an in situ polymerization method followed by hydrothermal treatment (Fig. 10a, b). The introduction of PANI can not only maintain the strong attachment between CuO nanorods and rGO surface, but also construct penetrating channels between CuO and rGO for rapid charge transfer. As a result, the CuO-PANI-rGO ternary hybrid displayed a high specific capacitance of 634.4 F g^−1^ at 1 A g^−1^ in an aqueous solution, and the self-assembled typical ternary hybrid device exhibited a high energy density of 126.8 Wh kg^−1^ at a striking power density of 114.2 kW kg^−1^. Besides, after 10,000 cycles, the specific capacitance lost only remained 2.6%, indicating an outstanding cycle life. In another article, a novel 3D porous graphene/polyaniline/Co_3_O_4_ ternary hybrid aerogel (3D GPC) was obtained by Lin and co-workers through a similar hydrothermal method [100]. The 3D GPC possessed an excellent electrochemical performance, including high specific capacitance (1247 F g^−1^ at 1 A g^−1^) and superior rate capability (755 F g^−1^ even at a current density of 20 A g^−1^). The energy density of 54.5 Wh kg^−1^ could be achieved at an ultrahigh power density of 3.8 kW kg^−1^ by calculation. The extraordinary capacitive properties of the 3D GPC hybrid can be explained by the following reasons: (1) the high pseudocapacitance contributed by the PANI and Co_3_O_4_; (2) the synergistic effect provided by the uniformly dispersed PANI/Co_3_O_4_ NPs onto graphene surface; (3) the effective diffusion paths constructed by the 3D porous structure. Zhang et al. prepared a series of ternary composites through the incorporation of NiO nanocrystals into PAN-based activated carbon nanofibers (ACNF/PANI/NiO) by the combination of multiple methods [77]. Due to the synergistic effect of NiO nanocrystals and PANI modified carbon substrates, the ACNF/PANI/NiO-0.3 (0.3 represents the mass of NiO) composite showed the highest specific capacitance 1157 F g^−1^ at a current density of 1 A g^−1^ in 6 M KOH electrolyte and still remained 720 F g^−1^ even at a high current density of 10 A g^−1^. Moreover, the ACNF/PANI/NiO-0.3 electrode-based asymmetric supercapacitor exhibited a superior energy density of 14.47 Wh kg^−1^ at a high power density of 651 W kg^−1^. Organic interlayer introduced between the carbon skeleton and TMCs that not only serves as a “bridge” for accelerating ion transfer in the interface but also provides a high pseudocapacitance, resulting in high capacitance and rate performance.

### Spatial Confinement

Besides, in order to construct an efficient binding interface between carbon skeletons and TMCs, the in situ spatial confinement effect also is a useful strategy in the preparation of TMCs/carbon composites [52, 101]. TMCs nanocrystals can be strongly confined in the pore or interlamination of carbon skeletons owing to the physical interaction between TMCs and carbon surface. The spatial confinement effect can not only prevent the emergence of direct detaching of TMCs from carbon surface during the long-term charge/discharge process, but also benefit the fast redox reaction kinetics based on the construction of electron transfer channels. Benefiting from the carbon skeletons with superior conductivity and mechanical strength, the charge transfer properties of TMCs/carbon composites can be largely improved, and the structural stabilities can also be correspondingly preserved, which exhibit great advantageous for the power/energy densities and long cycle life.

**Fig. 11:**
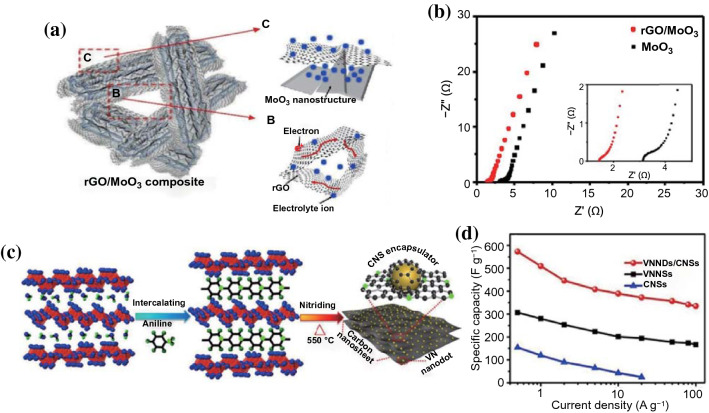
**a** Schematic illustration of the unique structure and synergetic effect of rGO/MoO_3_ composite. **b** Nyquist plot of the rGO/MoO_3_ composite and MoO_3_. Adapted with permission from Ref. [102] Copyright 2015 Wiley–VCH. **c** Schematic illustration of the preparation procedures of the VNNDs/CNSs. **d** Gravimetric capacitances of VNNDs/CNSs, VNNSs, and CNSs at different current densities. Adapted with permission from Ref. [78] Copyright 2018 Elsevier

For example, Cao et al. fabricated a porous rGO/MoO_3_ composite that the MoO_3_ particles were strongly wrapped by reduced graphene oxide (rGO) nanosheets through a simple mixture of Mo-MOFs and rGO sheets followed by an annealing process (Fig. 11a) [102]. As shown in Fig. 11b the conductivity of rGO/MoO_3_ composite was greatly improved in comparison to pristine MoO_3_, which mainly attributed to the unique structure and synergetic effect of rGO/MoO_3_ composite. The rGO network greatly reduced the impedance by providing a highly conductive and porous pathway for allowing fast electrons and ions transfer, which was beneficial for the improvement of rate performance. Moreover, the rGO film wrapping around MoO_3_ prevented the detaching and aggregation of MoO_3_ nanomaterials, greatly improving the superior cycling capability. Therefore, an all-solid-state flexible supercapacitor device based on rGO/MoO_3_ composite delivered a good energy density of 14 Wh kg^−1^ at a high power density of 500 W kg^−1^ and excellent capacitance retention of ≈ 80% after 5000 cycles at 2 A g^−1^.

Carbon nanosheets/VN nanodots (VNNDs/CNSs) hybrid electrode material was prepared by Li and co-workers via a facile spatially confined strategy that the VN nanodots (VNNDs) were intercalated into carbon nanosheets (CNSs) (Fig. 11c) [78]. The strategy can well disperse the VNNDs along with the formation of the pillared lamellar structure of CNSs and provide abundant active sites and enhance fast electrolyte ions/electrons diffusion kinetics. The VNNDs encapsulated in CNSs layers could effectively impede the electrochemical oxidation of VN and maintain a more stable structure during the longtime charge/discharge process. Compared with VNND or CNSs, the VNNDs/CNSs hybrid electrode exhibited an ultrahigh specific capacitance (573.1 F g^−1^ at 0.5 A g^−1^) and an excellent rate capability (the capacity retention of 58.4% at 100 A g^−1^ compared with that at 0.5 A g^−1^) (Fig. 11d). Moreover, the symmetric all solid-state flexible supercapacitors assembled by VNNDs/CNSs electrodes and KOH/PVA gel electrolyte showed an outstanding volumetric energy density of 16.1 Wh L^−1^ at an ultrahigh volumetric power density of 64,500 W L^−1^, proving a large potential in the next-generation energy storage devices high energy density.

Yang and his co-works successfully obtained Co_3_O_4_/P, N co-doped carbon composites (Co_3_O_4_@PNC) through confining the growth of Co_3_O_4_ nanoparticles within the nanopores of P, N co-doped carbon material [103]. Compared with Co_3_O_4_ (about 320 F g^−1^), the Co_3_O_4_@PNC-2 (2 represents mass loadings of Co_3_O_4_) composite showed a higher specific capacitance of 1310 F g^−1^ at 0.5 A g^−1^, which still remained at 655 F g^−1^ even at a high current density of 20 A g^−1^, indicating an excellent rate capability. This obvious improvement was mainly attributed that the introduction of carbon can not only enhance the conductivity of composite but also keep the uniform distribution of Co_3_O_4_ nanoparticles with ultrasmall size (2–5 nm). As a result, the asymmetric supercapacitor based on Co_3_O_4_@PNC-2//AC exhibited an ultra-high energy density of 47.18 Wh kg^−1^ at 375 W kg^−1^ and maintained at 34.22 Wh kg^−1^ even at an ultra-high power density of 7.5 kW kg^−1^.

As a kind method of spatial constraint, the design of core–shell structure also plays a crucial role in improving the electrochemical performance of TMCs [104]. Carbon shell with high mechanical strength not only could enhance the electrical conductivity of composite, but also protect against the degradation and aggregation of TMCs nanoparticles. In this case, Yoon et al. [79] designed and developed a novel millerite core-nitrogen-doped carbon hollow shell (NiS–NC HS) structure by using PD-derived carbon as coated and NiS as the core. Compared with pristine Ni_3_S_2_ (828.21 F g^−1^), the maximum specific capacitance of NiS–NC HS was 1170.72 F g^−1^ at 0.5 A g^−1^, and 90.71% of its initial capacitance could be maintained at 6 A g^−1^ after 4000 charge–discharge cycles. Moreover, the electrode still could retain 72.1% of its maximum capacitance even through the current density increasing to 10 A g^−1^, displaying a twofold higher rate capability compared with the pristine Ni_3_S_2_ (34.56%). In another article, a T-Nb_2_O_5_@carbon hollow core–shell nanostructures are constructed by Zhang et al. via using silica as a template [105]. The T-Nb_2_O_5_@carbon hollow core–shell nanostructures showed significant improvement in the rate capability (from 34 to 59%) compared to the T-Nb_2_O_5_ nanoparticles. More interestingly, hybrid supercapacitors based on the composite exhibited a stable cycle performance (85% capacitance retention after 10,000 cycles) and still processed an energy density of 12 Wh kg^−1^ at 16 kW kg^−1^. These results indicated that design of core–shell structure processed great constraining force for TMCs, in which carbon shell could not only enhance the whole conductivity for facilitating the electron transport, but also relieve the volume expansion of TMCs during the charge–discharge process, thereby ensuring the high rate capability and superior cycle stability.

Till now, despite the interface design of TMCs/carbon has already done a lot of research, achieving both outstanding energy density and superior power density of electrode materials is still a huge challenge, and the crucial challenge is to build up stable interface incorporation between carbon and TMCs. The combination of physical spatial constraints and chemical bonding may be a more efficient manner for achieving rapid electrons transfer at the interface of TMCs/carbon-based electrodes in the future.

## Regulation of TMC’s Electronic Structure

For most of TMCs, the regulation of electronic structure plays a crucial role in enhancing the electrochemical performance of supercapacitors [16, 106, 107]. For example, owing to the different interlink manners, manganese oxides exhibited abundant crystalline structures (including *α*, *β*, *γ*, *δ*, and *λ* forms), leading to different electronic structure [108]. Particularly, when the crystal size of TMCs is decreased to nanometer scale or quantum dots, the physical and chemical properties of such materials can make significant changes owing to some special effects such as small size effect, quantum tunneling effect, and surface effect. Moreover, doping heterogeneous atoms to adjust the crystal electronic structure followed by the synergistic effect of multi-metal can also further enhance the electrochemical performance [109, 110]. In recent years, the adjustment of TMC’s electronic structure to optimize TMCs/carbon electrodes has been investigated extensively for supercapacitors [16, 111, 112]. In this section, we will summarize the recent progress from the aspects of the effect of nano-crystallization or quantum dots construction, heterogeneous atoms doping, defects, and construction of heterogeneous interfaces in the regulation of TMC's electronic structure for TMCs/carbon-based supercapacitors with both high energy/power densities.

### Nano-Crystallization or Quantum Dots

**Fig. 12:**
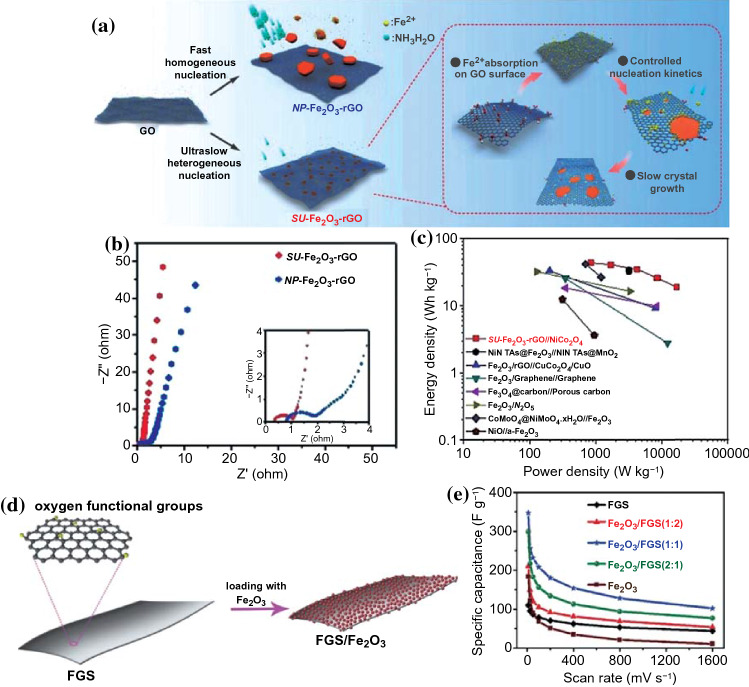
**a** Schematic illustration of the growth of SU-Fe_2_O_3_ sheets on graphene by means of controlled crystallization kinetics. **b** Comparison of electrochemical impedance of different size of Fe_2_O_3_-rGO. **c** Ragone plot related to the energy and power densities of SU-Fe_2_O_3_-rGO//NiCo_2_O_4_. Adapted with permission from Ref. [115] Copyright 2019 Wiley–VCH. **d** Schematic illustration of Fe_2_O_3_/FGS composite. **e** Specific capacitances of the pristine FGS, Fe_2_O_3_, and Fe_2_O_3_/FGS composite electrodes at different scan rate. Adapted with permission from Ref. [24] Copyright 2015 Wiley–VCH

Due to the sluggish reaction kinetics, poor electrical conductivity, and volumetric expansion effect, the high power densities and long cycle lives of TMC-based electrodes are greatly prevented in the practical application of supercapacitors. To solve the above challenges, the design of TMCs with nano-crystallization or quantum dots size is considered as an effective strategy [46]. Specifically, nano-structured TMCs can provide larger electrochemical active surfaces owing to surface effect, which shortens the ion diffusion distances and increases the contact areas with electrolytes [113, 114]. For example, Wang et al. [115] synthesized sub-nanometer, ultrafine a-Fe_2_O_3_ sheets loading on graphene (SU-Fe_2_O_3_-rGO) by controlling the crystallization kinetics (As shown in Fig. 11a). Compared with the NP-Fe_2_O_3_-rGO microparticles, the SU-Fe_2_O_3_-rGO with the ultrathin thickness (only about 0.9 nm) and smaller lateral size (about 14.5 nm) can avoid the slow ion diffusion kinetics in the bulk phase of Fe_2_O_3_ and greatly facilitate the ion/electron transport during electrochemical reactions (Fig. 12b). As a result, the SU-Fe_2_O_3_-rGO electrode exhibited a superior specific capacitance of 1575 F g^−1^ at 1.25 A g^−1^ and remained at 955 F g^−1^ even at a high current density of 25 A g^−1^, indicating a superior rate performance. When assembled as asymmetric supercapacitor using SU-Fe_2_O_3_-rGO as anode and NiCo_2_O_4_ hybrid as cathode, a high energy density of 43.6 Wh kg^−1^ could be achieved at a power density 844 W kg^−1^ (Fig. 12c) and the device possessed excellent cycle life with a capacitance retention of 106.4% after 30,000 cycles at 5 A g^−1^.

In a similar case, the GCFS-0.33 electrode with high specific capacitance (310.2 C g^−1^ at 2 mV s^−1^) was obtained in which the ternary Co_0.33_Fe_0.67_S_2_ nanoparticles (10–60 nm) were embedded between the graphene nanosheets through a facile one-step hydrothermal method [116]. When the GCFS-0.33 composites and sulfurized graphene/CoNiAl-layered double hydroxides were used as the negative and positive electrode, respectively, the as-fabricated ASCs exhibited a high energy density of 66.8 Wh kg^−1^ at a power density of 300.5 W kg^−1^ and still retained a high energy density of 13.1 Wh kg^−1^ even at an ultrahigh power density of 29.4 kW kg^−1^. Such a robust sandwich-like structure with superior mechanical integration and high electrical conductivity can provide unobstructed pathways for the fast diffusion/transportation of electrolyte ions/electrons during rapid charge/discharge processes, which is beneficial to improve the rate capability. Wang et al. [117] have synthesized a Ni_3_S_2_@Co_9_S_8_/N-HPC composite in which the Ni_3_S_2_ attaching Co_9_S_8_ nanocrystal was encapsulated into a 3D N-doped hierarchical porous carbon (N-HPC). The strawberry-like Ni_3_S_2_@Co_9_S_8_ nanoparticles (average diameter of 900 nm) confined in the carbon skeleton can provide high theoretical specific capacitance, and 3D interconnected hierarchical porous carbon framework can construct fast electrolyte ions/electrons transfer channels. Benefiting from the synergistic effect, the Ni_3_S_2_@Co_9_S_8_/N-HPC composite showed an ultrahigh specific capacitance and excellent cycling stability. In addition, the Ni_3_S_2_@Co_9_S_8_/N-HPC//HPC was assembled into an ASCs in a 6 M KOH electrolyte. The ASCs delivered a high energy density of 77.1 W h kg^−1^ at 263.3 W kg^−1^ and still remained as high as 36.1 Wh kg^−1^ even at an ultrahigh power density of 25.9 kW kg^−1^.

Quantum dots (QDs) have presented an exponential growth of research in various potential applications in the last decades, especially in supercapacitors [118–121]. Due to the quantum effect, TMCs-QDs as electrode materials for supercapacitors have received extensive attention [122]. For example, Xia et al. synthesized Fe_2_O_3_/FGS hybrid materials that Fe_2_O_3_ quantum dots (QDs) (about 2 nm) were uniformly decorated on the functionalized graphene sheets (FGS) through a thermal decomposition method (Fig. 12d) [24]. The Fe_2_O_3_ QDs can be evenly dispersed on the surface of FGS with a proper mass ratio of Fe_2_O_3_/FGS (2:1), and the high conductivity and large surface area of FGS efficiently suppress the agglomeration of Fe_2_O_3_ QDs. As a result, the asymmetrical supercapacitor based on Fe_2_O_3_/FGS exhibited a high energy density of 50.7 Wh kg^−1^ at a power density of 100 W kg^−1^ and cycling stability of 95% after 5000 cycles in a voltage window of 2 V. Moreover, a Nb_2_O_5_ QD-NC hybrid material was prepared by Liu et al. that Nb_2_O_5_ quantum dots (about 5 nm) were successfully embedded into the nitrogen-doped porous carbon (NC) derived from ZIF-8 dodecahedrons [123]. When assembled with commercial activated carbon cathode, the hybrid supercapacitors (HSCs) showed a high energy density of 76.9 Wh kg^−1^ at an ultrahigh power density of 11,250 W kg^−1^ and superior cycling stability (about capacity retention of 85%) in an organic condition. The excellent performance of Nb_2_O_5_ QD-NC can be ascribed to the following reasons: (1) the high surface area and highly porous structure of NC can provide more ion attachment site which is beneficial for the fast electrolyte ions/electrons transfer; (2) ultrasmall size (ca. 5 nm) may shorten the ion diffusion and electron transportation distance; (3) the uniform distribution of Nb_2_O_5_ QDs and strongly interfacial binding between Nb_2_O_5_ QDs and NC can prevent the volume change upon cycling; (4) N doping may boost the electronic properties and provide additional charge-storage sites.

In contrast to conventional bulk materials, TMCs with nanoscale size can greatly improve ionic and electronic transport, which may be a promising solution for the achievement of both high energy and power densities for TMCs/carbon-based supercapacitors [124]. Consequently, nanoscale TMCs crystals supported by the conductive carbon skeletons can not only avoid the deficiency of single material, but also greatly boost the practical application of TMCs/carbon electrodes in energy storage devices.

### Heterogeneous Atoms Doping

**Fig. 13:**
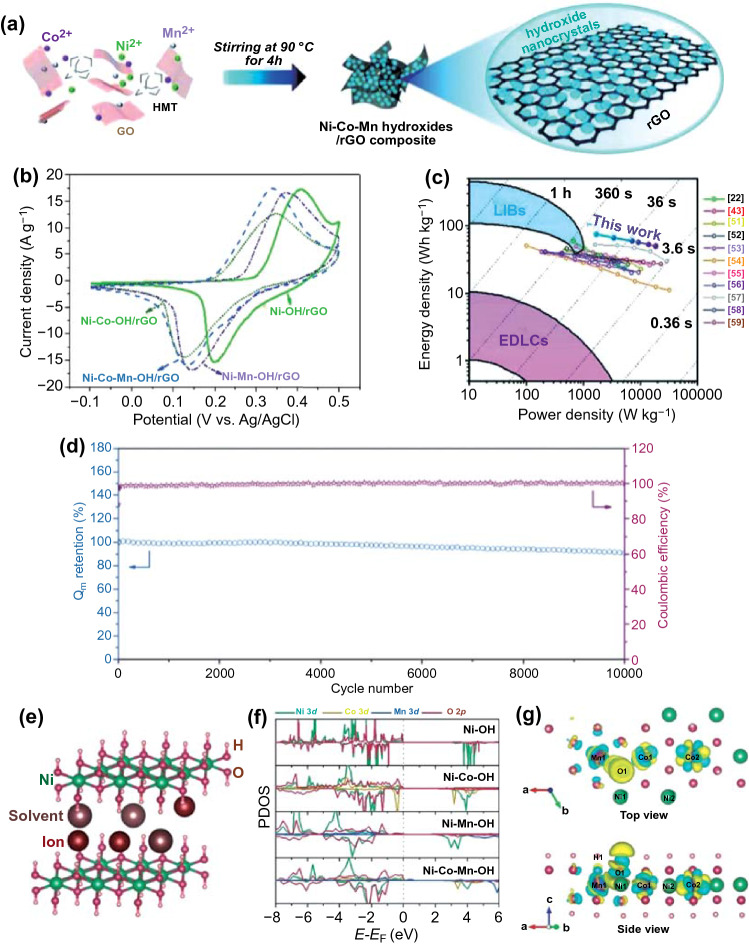
**a** Schematic illustration of the preparation of Ni–Co–Mn–OH/rGO. **b** CV curves at 5 mV s^−1^. **c** Ragone plot of the Ni–Co–Mn–OH/rGO//PPD/rGO hybrid supercapacitor device. **d** Cycling performance of the hybrid supercapacitor device at 20 A g^−1^. **e**–**g** Schematic illustration of nickel hydroxide-layered structure with interlayer species and two possible mechanisms to store charge and density of state (PDOS) diagrams. Adapted with permission from Ref. [130] Copyright 2018 Wiley–VCH

Heterogeneous atoms doping is another effective strategy, which can fundamentally regulate the electronic structure and alter the electron distribution to further improve the reaction activity and the electrochemical performance of TMCs/carbon based supercapacitors [125, 126]. According to the doping type, heterogeneous atoms doping can be divided into cation doping (heterogeneous metal atoms, e.g., Ni and Co, Co and W, Mn and Fe, even more, metal atoms, etc.) and anion doping (e.g., N, P, or F). Owing to the synergistic effect of the multiple transition metal atoms, combination of various TMCs can provide highly electrochemical conductivity and abundant redox activity, which is beneficial for the electrochemical performance [127, 128, 129]. For example, Liu’s group systematically studied the influence of cobalt and manganese atoms (Co, Mn) on the single doping or co-doping of Ni(OH)_2_ crystals by DFT calculations [130]. The Mn doping could increase the capacity owing to the lower deprotonation energy and facile electron transfer path, while the Co doping could improve the structural stability of the entire electrode (Fig. 13e–g) [130]. As shown in Fig. 13b-d, the Ni–Co–Mn–OH/rGO//PPD/rGO hybrid capacitor demonstrated remarkable energy densities of 74.7 and 49.9 W kg^−1^ at the power densities of 1.68 and 18.5 kW kg^−1^, respectively. Moreover, 91% of its initial capacity could be retained after 10,000 cycles at 20 A g^−1^, showing super-fast energy storage capacity and long cycle life. In another article, Mohamed et al. [131] fabricated Co-W-S composites in an N, S co-doped porous carbon matrix (Co-W-S@N, S-PC) using PTA@ZIF-67 as a precursor via a simple carbonization method. Compared with the Co_1−*x*_S@N, S-PC, the introduction of W atom could not only increase the redox activity of transition metal redox centers, but also provide additional faradaic pseudocapacitance. As a result, the Co-W-S@N, S-PC electrode demonstrated an ultrahigh specific capacitance (1929 F g^−1^ at 5 mV s^−1^). Furthermore, the asymmetric supercapacitor device based on Co-W-S@N, S-PC//AC delivered a superior energy density of 32.9 Wh kg^−1^ at the high power density of 700.2 W kg^−1^ and good cyclic stability with capacitance retention of 77% after 5000 cycles at 10 A g^−1^.

In contrast to cation doping, the anion doping of TMCs based electrodes is rarely reported. The experimental results and DFT calculations have confirmed that the anion-doping in TMCs could act as an effective approach to enhance the electrochemical conductivity and improve the reaction kinetics [53, 132]. For example, Liu et al. developed a P-CoS_2_@P, S, N–C hybrid of P-doped CoS_2_ (P-CoS_2_) nanoparticles confined in highly conductive P, S, N tri-doped carbon skeleton (P, S, N–C) [133]. The P-CoS_2_@P, S, N–C hybrid delivered a high specific capacity of 689 C g^−1^ at 2 A g^−1^, and still possessed capacitance retention of 77.5% even at a high current density of 30 A g^−1^. This was mainly because the introduction of P atom could enhance the covalency and decrease the migration energy of electrons, which largely boosted the electrochemical conductivity. In addition, ultrafine P-Co_3_O_4_ nanoparticles embedded into P, N–C nanowires (P-Co_3_O_4_@P, N–C) were also prepared via an in situ structural reconstruction strategy [53]. The theoretical calculation and experimental results showed that P doping could improve the electrochemical reactivity and facilitate the electrons/ions transport. Therefore, the appropriate incorporation of a small number of heterogeneous atoms into TMCs can not only provide additional capacity but also improve its electrical conductivity, which provides a helpful way to regulate the electronic structure of the TMCs.

### Defects

Defects, such as vacancies, edges, grain boundaries, substitutional impurities, and so on, always play significant roles in improving geometric and electronic structures of TMCs [134]. Different defects often play different functions in improving the electrochemical performance of TMCs, such as vacancies increasing more active sites and enhancing conductivity [135, 136], substitutional impurities providing additional capacitances and so on [131, 137]. Specifically, the vacancies including metal vacancies and nonmetallic vacancies have been extensively explored to accelerate the intrinsic conductivity and redox activity of TMCs for supercapacitors [136, 138, 139]. In this part, taking oxygen vacancies, for example, we will mainly discuss the effect of oxygen vacancies on the electrochemical performance of transition metal oxides (TMOs) based supercapacitors.

**Fig. 14:**
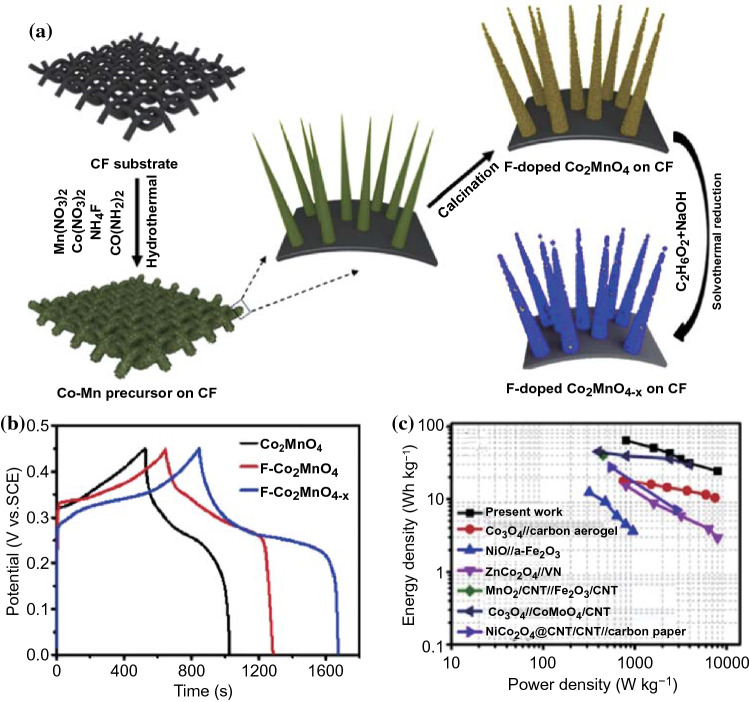
**a** Schematic of the synthesis of F-Co_2_MnO_4−x_ nanowires on CF substrate. **b** GCD curves of Co_2_MnO_4_, F-Co_2_MnO_4_, and F-Co_2_MnO_4−*x*_ electrodes at 1 A g^−1^. **c** Ragone plot of F-Co_2_MnO_4−*x*_ /CF//Fe_2_O_3_/CF device. Adapted with permission from Ref. [125] Copyright 2019 Elsevier

Kim et al. have confirmed that the presence of oxygen vacancies in α-MoO_3_ can greatly improve the electrical conductivity (electrical conductivity from 10^–5 ^S cm^−1^ increasing to 10^–4^ S cm^−1^) and broaden the interlayer spacing. The experimental results showed that MoO_3–*x*_ had higher capacity (∼ 550 C g^−1^) and better cycling stability (76% after 10,000 cycles) compared with MoO_3_ (< 400 C g^−1^, 50% after 50 cycles) at a sweep rate of 100 mV s^−1^ [140]. The incorporation of oxygen vacancies could act as electron donors and consequently increase the free carrier concentration as well as provide more exposed electrochemical active sites, resulting in improved electrochemical properties [141]. To gain abundant oxygen vacancies, Yang and co-workers firstly prepared ultrafine Co_3_O_4_ nanoparticles/graphene (UCNG) composites with abundant oxygen-vacancy via a new-type one-step laser irradiation strategy [142]. The theoretical calculations demonstrated that the surface oxygen vacancies could promote the electrons transfer by creating midgap electronic states. As a result, the UCNG electrode achieved a high specific capacitance of 978.1 F g^−1^ at 1 A g^−1^ and more than capacitance retention of 93.7% even at a high current density of 10 A g^−1^. Moreover, based on the fluorine-doped, Liu et al. [125] successfully fabricated F-Co_2_MnO_4_-x/CF composite by introducing oxygen vacancies into Co_2_MnO_4_ (Fig. 14a). As advanced electrode materials for supercapacitor, the F-Co_2_MnO_4−*x*_/CF exhibited a higher specific capacity of 269 mAh g^−1^ and more superior cyclic stability (93.3% of the initial specific capacity after 5000 cycles) in comparison to the F-Co_2_MnO_4_/CF (204 mAh g^−1^, 90.9%) and Co_2_MnO_4_/CF (165 mAh g^−1^, 85.8%), principally demonstrating that the introduction of F dopants and oxygen vacancies could synergistically increase the electrical conductivity and reactivity of electrochemically active sites (Fig. 14b). Moreover, the ASCs of F-Co_2_MnO_4−*x*_ /CF//Fe_2_O_3_/CF exhibited high energy density of 64.4 Wh kg^−1^ at a power density of 800 W kg^−1^ (Fig. 14c).

In short, the introduction of oxygen vacancies into TMOs can tremendously improve the electrochemical performance for supercapacitors, e g., greater electrical conductivity, more prominent specific capacitance, rate performance, and cyclic stability. Besides, the distribution of the defects is also a critical factor of the electrochemical performance enhancement, oxygen vacancies with uniform distribution in TMCs are beneficial to improve the cycle stability [143]. Therefore, defects reasonably introduced into electrode materials can not only contribute to improving the physicochemical properties but also adjust the performance of materials.

### Heterogeneous Interface

**Fig. 15:**
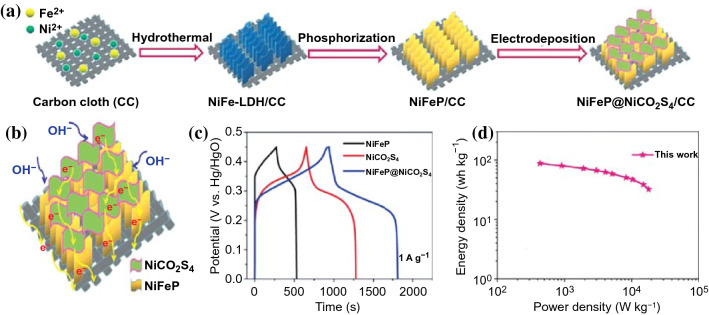
**a** Schematic of the NiFeP@NiCo_2_S_4_ hybrid nanosheets synthesis process. **b** Schematic description of charge storage and transfer advantages of NiFeP@NiCo_2_S_4_/CC electrode. **c** GCD of NiFeP/CC, NiCo_2_S_4_/CC, and NiFeP@NiCo_2_S_4_/CC electrodes at 1 A g^−1^. **d** Ragone plot of the e NiFeP@NiCo_2_S_4_/CC//OPC-850 ASCs and a comparison with previous works. Adapted with permission from Ref. [149] Copyright 2020 Elsevier

In the past few years, the construction of heterogeneous interfaces during the synthesis of TMCs crystals has aroused extensive research interests due to its improved high ion carrier mobility and broad electrode potential, mainly because the unique structure can decrease the activity of hydrogen evolution reaction (HER) and oxygen evolution reaction (OER) at high voltage window [144–147]. For example, the NiFeP@NiCo_2_S_4_/carbon cloth (NiFeP@NiCo_2_S_4_/CC) hybrid electrode with NiFeP@NiCo_2_S_4_ heterostructure was successfully manufactured by using a novel combination of hydrothermal reaction, phosphorization treatment, and electrodeposition technique strategy (Fig. 15a) [148]. Compared with NiFeP/CC and NiCo_2_S_4_/CC, the NiFeP@NiCo_2_S_4_/CC hybrid electrode demonstrated higher specific capacitance (Fig. 15c), better rate capability, and more excellent cycling stability. In an asymmetric supercapacitor of NiFeP@NiCo_2_S_4_/CC//porous carbon OPC-850, the device obtained a superior energy density of 32.1 Wh kg^−1^ at an ultrahigh power density of 18,034.2 W kg^−1^ (Fig. 15d). The distinguished electrochemical performance can be attributed that the unique heterostructure not only provides abundant open channels to achieve rapid electronic transport, but also enlarge accessible 2D surface to offer more active sites for absorbing electrolyte ions (Fig. 15b).

In addition, bimetallic MOF-derived NiCo_2_S_4_-Ni_9_S_8_-C double-layered yolk–shell microspheres were synthesized by Yan et al. [149]. Owing to the existence of NiCo_2_S_4_-Ni_9_S_8_ hetero-interface, the composite materials exhibited superior specific capacity (293.6 mAh g^−1^ at 1 A g^−1^) and good rate ability (capacitance retained 81.1% as current densities increased from 1 to 20 A g^−1^). Based on DFT calculations, the authors also confirmed that the hetero-interfaces of NiCo_2_S_4_-Ni_9_S_8_ changed the electronic distribution, thus strengthening the electrochemical conductivity of the entire electrode. Moreover, the hybrid supercapacitors assembled by NiCo_2_S_4_-Ni_9_S_8_-C DYMs and graphene hydrogel showed an excellent energy density of 51 Wh kg^−1^ at a superhigh power density of 1399.4 W kg^−1^. In another article, Dang and his co-workers fabricated a 3D NiCoP/CoP hetero-nanosheets network supported by N-CNTs (N-CTs@NiCoP/CoP) via one-step phosphorization of nickel/cobalt hydroxide [150]. The hybrid supercapacitor of N-CNTs@NiCoP/CoP//ZIF-67-derived porous carbon demonstrated an outstanding energy density of 45.5 Wh kg^−1^ at 784 W kg^−1^ and great stability of 87% retention (after 10,000 cycles at 12 A g^−1^). The superior electrochemical performance can be mainly ascribed to the strong synergistic effect of NiCoP/CoP heterojunctions, the large surface area of N-CNTs skeletons, and 3D conductive networks.

In conclusion, a heterogeneous interface consisting of two or more types of TMCs materials not only enhances the structural stability, but also provides fast pathways for electrolyte ion/electrons transfer, leading to the improved electrochemical performance of the whole electrode, which could offer a good thought of a structural design for TMCs/carbon based supercapacitors.

## Summary and Outlook

TMCs/carbon-based supercapacitors with the potential high energy densities represent the application tendency of next-generation energy storage systems. However, as for the practical and large-scale applications, the poor power density and inferior cycle stability derived from the low intrinsic conductivity and structural instability of TMCs are critical constraints. In this review, focusing on the current fundamental understanding, challenges, and opportunities, we systematically discuss the possible design strategies for the TMCs/carbon-based supercapacitors from the aspects of conductive carbon substrates, interface engineering, and modification of electronic structures for the first time. Firstly, we present the structural design strategies for the carbon skeletons with different dimensions. As the electrode supports and conductive skeletons, carbon substrates with high electron conductivities to guarantee the rapid charge transfer, abundant pore channels to promote the electrolyte ions migration, large specific surface area to load more amounts of TMCs, even superior mechanical stability/flexibility to support long cycle life, etc., are greatly needed. Secondly, we point out that the strong interfaces between carbon skeletons and TMCs play a crucial role in achieving high power density and long cycle stability of TMCs/carbon-based supercapacitors. Specifically, we describe four feasible manners for the construction of steady interfaces between carbon skeletons and TMCs by the modification of carbon surface or physical interaction, including heteroatom-doping, functional groups modification, organic interlayer introduction, and spatial confinement. Lastly, the modification of TMCs (including TMCs-QDs) crystals with nanoscale dimensions can reduce the electron/ion diffusion distance, increase the electrode/electrolyte contact surface, improve the intrinsic conductivity/redox activity, etc., and also have been presented as one guarantee role to realize both high power/energy densities of TMCs/carbon based supercapacitors.

Despite large amounts of research that have been done to study the assemble strategies, electrochemical performance, and energy storage mechanism of TMCs/carbon based supercapacitors, some critical challenges still need to be considered: (1) How to achieve the high-efficiency utilization of carbon as conductive support plays a pretty important role. Although large specific surface area and porous structure of carbon substrate can provide high conductivity and abundant electrons/electrolyte ions transfer channels, carbon materials with low specific capacitance restrict its development. Therefore, the high-efficiency utilization of carbon as conductive substrate needs to be considered on basis of appropriate mass ratio between carbon and TMCs, achieving high energy density as well as high power density. (2) The trade-off between high mass loading and agglomeration of nanoscale TMCs crystals also is a challenge. TMCs with nanostructure can achieve high mass loading and efficient utilization ratio, which possess much higher faradaic pseudocapacitive reaction kinetics. However, in a real scenario, the high surface energy of TMCs with ultra-small sizes is easy to be agglomerated, resulting in sluggish reaction kinetics as same as bulk materials. (3) New techniques and methods are urgently needed to construct steady composite interfaces. For composite materials, the fast transfer of electrolyte ions/electrons across the heterogeneous interfaces plays an important role in achieving ultra-high charge/discharge rates. However, how to build a steady composite interface is still a problem that needs deep thinking. (4) Besides, the choices of electrolyte, current collector, separator, and other components are also equally important. Notably, the form of free-standing film electrodes attracted more and more attention owing to no additional binders, conductors, or collectors. Compared with powder electrodes, it can increase mass loading of active materials, simplify the electrode preparation process, and avoid uncontrollable side reactions, which may be an important development direction of energy storage industry in the future.

This review provides a comprehensive and in-depth discussion about the structural design strategies for the high power TMCs/carbon-based supercapacitors. We try to present the classic and latest research findings and propose progressive perspectives towards the critical challenges that are relevant to the practical and large-scale application of TMCs/carbon-based supercapacitors. Overall, it is highly expected that more novel design strategies and efficient TMCs/carbon-based supercapacitors could come into the picture and further revolutionize the way we live in the future.
